# Degradation of RNA during lysis of *Escherichia coli* cells in agarose plugs breaks the chromosome

**DOI:** 10.1371/journal.pone.0190177

**Published:** 2017-12-21

**Authors:** Sharik R. Khan, Andrei Kuzminov

**Affiliations:** Department of Microbiology, University of Illinois at Urbana-Champaign, Urbana, Illinois, United States of America; Florida International University, UNITED STATES

## Abstract

The nucleoid of *Escherichia coli* comprises DNA, nucleoid associated proteins (NAPs) and RNA, whose role is unclear. We found that lysing bacterial cells embedded in agarose plugs in the presence of RNases caused massive fragmentation of the chromosomal DNA. This RNase-induced chromosomal fragmentation (RiCF) was completely dependent on the presence of RNase around lysing cells, while the maximal chromosomal breakage required fast cell lysis. Cell lysis in plugs without RNAse made the chromosomal DNA resistant to subsequent RNAse treatment. RiCF was not influenced by changes in the DNA supercoiling, but was influenced by growth temperature or age of the culture. RiCF was partially dependent on H-NS, histone-like nucleoid structuring- and global transcription regulator protein. The *hupAB* deletion of heat-unstable nucleoid protein (HU) caused increase in spontaneous fragmentation that was further increased when combined with deletions in two non-coding RNAs, *nc1* and *nc5*. RiCF was completely dependent upon endonuclease I, a periplasmic deoxyribonuclease that is normally found inhibited by cellular RNA. Unlike RiCF, the spontaneous fragmentation in *hupAB nc1 nc5* quadruple mutant was resistant to deletion of endonuclease I. RiCF-like phenomenon was observed without addition of RNase to agarose plugs if EDTA was significantly reduced during cell lysis. Addition of RNase under this condition was synergistic, breaking chromosomes into pieces too small to be retained by the pulsed field gels. RNase-independent fragmentation was qualitatively and quantitatively comparable to RiCF and was partially mediated by endonuclease I.

## Introduction

In bacteria, chromosomes exist in a condensed, poorly defined mass called the nucleoid. Bacterial DNA lacks histones and the higher-level chromatin organization of the eukaryotic chromosomes, and is confined in the cytosolic space by molecular crowding [1–3]. The structure of nucleoid is maintained by DNA condensation, achieved mostly by unconstrained DNA supercoiling and by DNA bending and looping action of the nucleoid-associated proteins (NAPs) [4]. In *Escherichia coli*, a total of about 200 different proteins are associated with the nucleoid [5], and their composition and copy number changes during growth, especially upon entry into the stationary phase. Out of these proteins, several major NAPs have been characterized in detail and include histone-like nucleoid structuring protein (H-NS), integration host factor (IHF), heat-unstable nucleoid protein (HU), factor for inversion stimulation (Fis), suppressor of T4 *td* mutant phenotype A (StpA), and DNA-binding protein from starved cells (Dps). These proteins, present in thousands of copies per cell at particular growth phases, participate in major cellular activities that include transcription, translation, replication and DNA condensation [6–7].

Besides the nucleoid-associated proteins, unknown species of RNA have also been found associated with purified nucleoids [2,8–12], contributing to their compactness. Identity of these RNA, however, was largely unknown until recently, when several small RNA molecules associated with the NAP HU were shown to contribute to the nucleoid structure [12,13].

**Fig 1:**
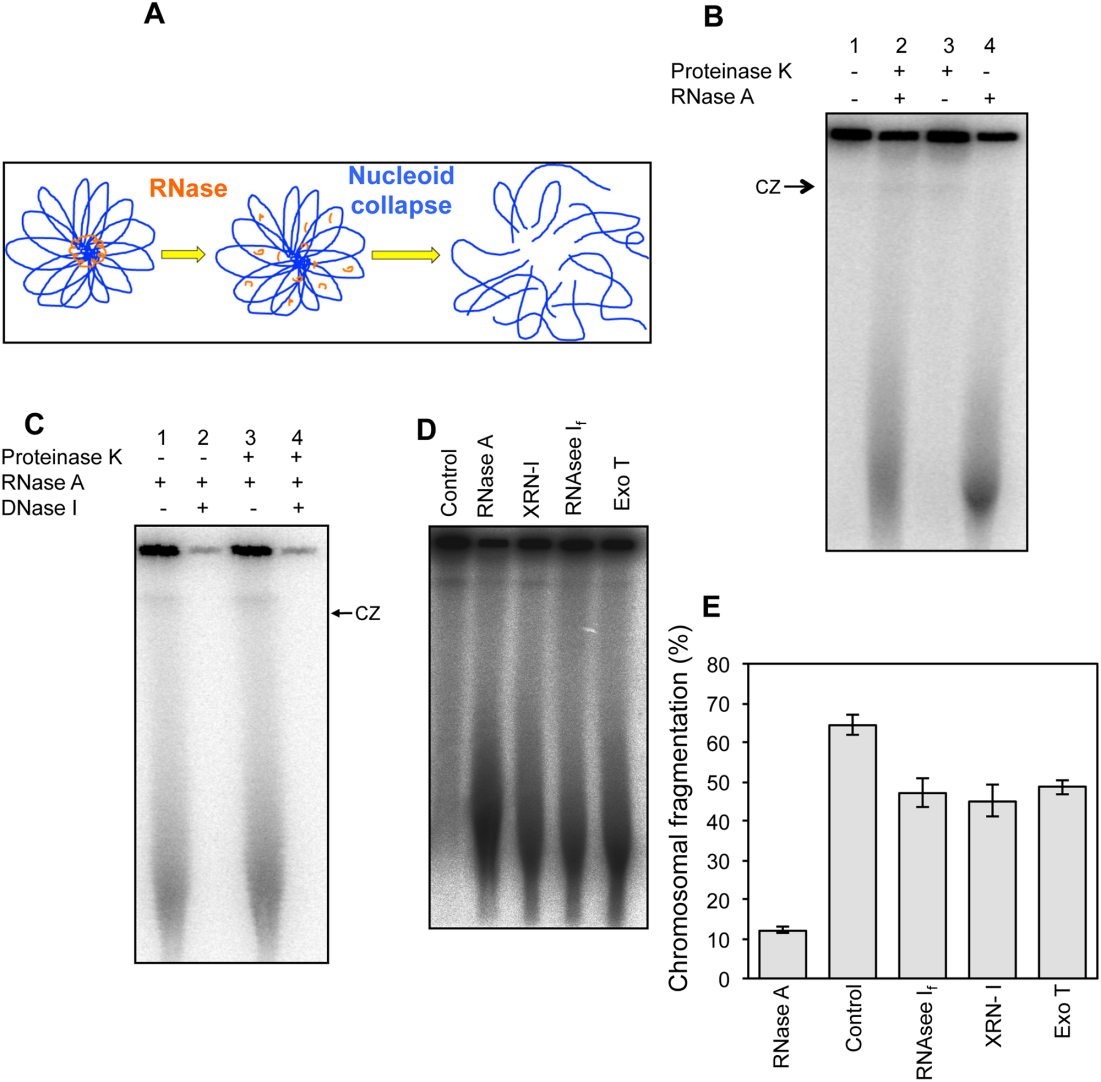
RNA degradation causes chromosomal fragmentation. **(A)** Schematics of a hypothetical scenario when RNA makes the central core of nucleoids, and its degradation results in collapse of the nucleoid structure, causing chromosomal fragmentation. **(B)** Radiogram of a pulsed field gel showing chromosomal fragmentation in AB1157 when cells were embedded in agarose plugs in the presence and absence of proteinase K (25 μg/plug) and/or RNase (50 μg/plug) and lysed overnight at 62°C. **(C)** Radiogram showing DNase I sensitivity of the signal entering the gel. Plugs were lysed at 62°C, washed extensively to remove traces of lysis buffer and then treated with DNase I at 37°C before PFGE. **(D)** A representative gel showing that RNA degradation by different enzymes causes chromosomal fragmentation. Plugs were made in the absence of proteinase K in 1x restriction enzyme buffer (NEBuffer 3 for RNase A, XRN-1 and RNAse I_f_ and NEBuffer 4 for Exo T). The concentrations of the enzymes used were, RNase, 50 μg/plug; XRN-1, 5 U/plug; RNAse I_f_, 100 U/plug and Exo T, 20 U/plug. **(E)** Quantification of the chromosomal fragmentation when plugs were made in the presence of various RNA degrading enzymes. The values presented are means of four independent assays ± SEM. CZ, compression zone.

We have been investigating how DNA damage, generated due to defects in the DNA metabolism or exposure to clastogenic agents, affects chromosome breakage [14–19]. We quantify double-strand breaks (DSBs) in chromosomal DNA by lysing radiolabeled *E*. *coli* cells in agarose plugs and separating the released circular chromosomes from linear subchromosomal fragments with pulsed field gel electrophoresis (PFGE) [16–17]. While standardizing the conditions of cell lysis and PFGE, we discovered massive chromosomal fragmentation when ribonuclease A (RNase A) was present during cell lysis within agarose plugs. While RNase-mediated relaxation of the released nucleoids was expected on the basis of reports of the critical role of RNA in the nucleoid structure or stability [9,10] (Fig 1A), generation of chromosomal breaks by RNase treatment was both novel and unexpected.

In this report, we show that RNase-induced chromosomal fragmentation (RiCF) is fast and dynamic and is influenced by a number of factors including growth and cell lysis parameters, but is resistant to inhibition of transcription or translation. We also show that RiCF is completely dependent upon endonuclease I, an enzyme reported to be compartmentalized in the periplasmic space, while the maximal RiCF requires the presence of at least one NAP, H-NS. Finally, we report spontaneous chromosomal breaks in *hupAB* double and *hupAB nc1 nc5* quadruple mutant, suggesting that HupA and HupB proteins, together with the non-coding RNAs *nc1* and *nc5*, are required for nucleoid stability.

## Materials and methods

### Bacterial strains, plasmids, growth conditions and reagents

All *E*. *coli* strains used in this study are derivatives of K-12 and are described in Table 1. All strains were grown in LB (10 g tryptone, 5 g yeast extract, 5 g NaCl per 1 L of H_2_O, pH to 7.4 with 250 μl 4 M NaOH; LB agar in addition contained 15 g agar per 1 liter of LB) at either 28°C or 37°C, unless stated otherwise. When required, antibiotics were added to the following final concentrations in μg/ml: ampicillin, 100; kanamycin, 50; chloramphenicol, 10 or 30; tetracycline, 10. Alleles were moved among strains by P1 transduction, and mutants were confirmed by polymerase chain reactions (PCR) or by functional tests including characteristic UV-resistance and temperature sensitivities. The *ΔhupB*, *ΔihfB* and *Δhns* mutations were created by replacing the genes with the *cat* cassettes using deletion-insertion system of Datsenko and Wanner [20]. The *nc1* and *nc5* deletions were created likewise by replacing the intergenic regions between *intF*-*yagP* and *yjdN*-*yjdM* (*phnB*-*phnA*) [12] with the *kan* and *cat* cassettes, respectively. Ribonuclease A (RNase A) was purchased from Boehringer Mannheim or Sigma, and its stock solutions were made as described in Molecular Cloning (2^nd^ edition). Briefly, RNase A was dissolved in 10 mM Sodium acetate (pH 5.2) to a final concentration of 10 mg/ml and incubated in boiling water for 15 min to inactivate any residual DNase activity. The pH of the solution was adjusted by addition of 1/10^th^ volume of 1M Tris HCl (pH 7.4), and the stock was diluted to a final concentration of 2 mg/ml and stored at –20°C. Proteinase K was from Roche Applied Science. XRN-I, RNase I_f_, Exonuclease T (Exo T), EcoRI and RNase A inhibitor were all from New England Biolabs.

**Table 1:** Strains used in this study.

Strains	Relevant characteristics	References / sources
AB1157	wild-type strain	[21]
JW3964-1	BW25113 Δ*hupA771*::*kan*	CGSC
JW1702-1	BW25113 Δ*ihfA786*::*kan*	CGSC
JW3229-1	BW25113 Δ*fisA779*::*kan*	CGSC
JW2644-3	BW25113 Δ*stpA750*::*kan*	CGSC
JW 0603–2	BW25113 Δ*rna749*::*kan*	CGSC
JW 2912–1	BW25113 Δ*endA720*::*kan*	CGSC
JM83Δ*rnr*::kan	F^−^*ara*Δ*lac-proAB*) *rpsL*(*str*^*r*^) Δ*rnr*::kan	[22]
JM83*Δpnp*::kan	F^−^*ara*Δ(*lac-proAB*) *rpsL*(*str*^*r*^) Δ*pnp*::kan	[22]
AK11	AB1157 *gyrB*(TS)	Lab Collection
SRK254	AB1157 Δ*hns*::*cat*	This Study
SRK254-ML	AB1157 Δ*hns*	This Study
SRK254-1	AB1157 Δ*ihfA786*::*kan*	AB1157 x P1 JW1702-1
SRK254-2	AB1157 Δ*ihfB*::*cat*	This Study
SRK254-3	AB1157 Δ*ihfA786*::*kan* Δ*ihfB*::*cat*	SRK254-1 x P1 SRK254-2
SRK254-4	AB1157 Δ*hupA771*::*kan*	AB1157 x P1 JW3964-1
SRK254-5	AB1157 Δ*hupB*::*cat*	This Study
SRK254-6	AB1157 Δ*hupA771*::*kan* Δ*hupB*::*cat*	SRK254-4 x P1 SRK254-5
SRK254-7	AB1157 Δ*nc1*::*kan*	This Study
SRK254-8	AB1157 Δ*nc5*::*cat*	This Study
SRK254-9	AB1157 Δ*nc1*::*kan* Δ*nc5*::*cat*	SRK254-7 x P1 SRK254-8
SRK254-10	AB1157 Δ*nc1* Δ*nc5*	This Study
SRK254-11	AB1157 Δ*nc1* Δ*nc5* Δ*ihfA*::*kan*	SRK254-10 x P1 JW1702-1
SRK254-12	AB1157 Δ*nc1* Δ*nc5* Δ*ihfA786*::*kan* Δ*ihfB*::*cat*	SRK254-11 x P1 SRK254-2
SRK254-13	AB1157 Δ*nc1* Δ*nc5* Δ*hupA771*::*kan*	SRK254-10 x P1 JW3964-1
SRK254-14	AB1157 Δ*nc1* Δ*nc5* Δ*hupB*::*cat*	SRK254-10 x P1 SRK254-5
SRK254-15	AB1157 Δ*nc1* Δ*nc5* Δ*hupA771*::*kan* Δ*hupB*::*cat*	SRK254-13 x P1 SRK254-5
SRK254-15-I	AB1157 Δ*nc1* Δ*nc5* Δ*hupAB*	This Study
SRK254-16	AB1157 Δ*hns* Δ*nc1*::*kan*	SRK254-ML x P1 SRK254-7
SRK254-17	AB1157 Δ*hns* Δ*nc5*::*cat*	SRK254-ML x P1 SRK254-8
SRK254-18	AB1157 Δ*hns* Δ*nc1*::*kan* Δ*nc5*::*cat*	SRK254-16 x P1 SRK254-8
SRK254-19	AB1157 Δ*fisA779*::*kan*	AB1157 x P1 JW3229-1
SRK254-20	AB1157 Δ*stpA750*::*kan*	AB1157 x P1 JW2644-3
SRK254-21	AB1157 Δ*pnp*::*kan*	AB1157 x P1 JM83 Δ*pnp*::*kan*
SRK254-22	AB1157 Δ*rnr*::*kan*	AB1157 x P1 JM83 Δ*rnr*::*kan*
SRK254-23	AB1157 Δ*endA720*::*kan*	AB1157 x P1 JW2912-1
SRK254-24	AB1157 Δ*rna749*::*kan*	AB1157 x P1 JW0603-2
SRK254-24-I	AB1157 Δ*endA*	This Study
SRK254-25	AB1157 Δ*endA* Δ*pnp*::*kan*	SRK254-24-I x P1 JM83 Δ*pnp*::*kan*
SRK254-26	AB1157 Δ*endA* Δ*rnr*::*kan*	SRK254-24-I x P1 JM83 Δ*rnr*::*kan*
SRK254-27	AB1157 Δ*nc1* Δ*nc5* Δ*hupAB* Δ*endA720*::*kan*	SRK254-15-I x P1 JW2912-1
SRK254-28	AB1157 Δ*hns*::*cat* Δ*rna749*::*kan*	SRK254 x P1 JW0603-2
SRK254-29	BW25113 Δ*rna749*::*kan* Δ*hns*::*cat*	JW0603-2 x P1 SRK254

### Radiolabeling and lysis of cells in agarose plugs

Cells were radiolabeled by diluting saturated cultures 100-fold in fresh LB containing 0.5–2 μCi/ml of ^32^P-orthophosphoric acid (MP Biomedicals or Perkin-Elmer) and growing them aerobically at 37°C until A_600_ of 0.6–1.0. At this time, 0.5–1.0 ml cultures were harvested by centrifugation, and cell pellets were suspended in 60 μl of TE buffer (10 mM Tris-HCl, 1 mM EDTA, pH 7.4). To make agarose plugs, 5 μl of proteinase K (from 5 mg/ml stock) and 65 μl of molten and cooled to 75°C agarose (either plain or lysis-plug agarose) was added to the cell suspension, and the mixture was quickly transferred to plug molds (Bio-Rad). Standard lysis plug agarose contained .2x of lysis buffer (1.2% agarose in .2% lauroyl sarcosine, 10 mM Tris-HCl, 5 mM EDTA, pH 8.0); any deviations in composition of plug agarose are described in individual assays. To determine the effect of RNAse A, cell pellets were suspended in 35 μl (40 μl in plugs made without proteinase K) of sterile TE buffer, and plugs were made immediately after adding 5 μl of proteinase K (from 5 mg/ml stock), 25 μl of RNase A (2 mg / ml stock) and molten plain or lysis-plug agarose, as described in experiments. Concentration of RNase A in plugs was varied by changing the amounts of RNase A and TE to keep the final volume of cell suspensions (before plug agarose addition) at 65 μl. Conditions used for making plugs with other RNases—XRN-I, RNase I_f_, and Exo T,–are described in the corresponding assays. The plugs were allowed to solidify at room temperature for 2–5 minutes and then transferred from the molds into glass tubes containing 1 ml of the 1 x lysis buffer (1% lauroyl sarcosine, 50 mM Tris-HCl, 25 mM EDTA, pH 8.0). The tubes were incubated in a 60–65°C water bath for 14–18 hours. Any variations in the composition of lysis buffer, as well as conditions of plugs lysis, are described in individual experiments.

### Pulsed-field gel electrophoresis

The lysed plugs were loaded onto 1% agarose gel made in 0.5x Tris-Borate-EDTA (TBE) buffer and electrophoresed at 6 V/cm for 20–24 hours using initial and final switch times of 60 and 120 seconds, respectively, in Bio-Rad CHEF-DRII PFGE system. The gel was dried under vacuum, exposed to a PhosphorImager screen and scanned in FLA-3000 series fluorescent image analyzer (FujiFilm). The data were processed using Image Gauge v3.41 software (Fuji). The percent of chromosomal fragmentation was calculated as the signal in the lane below the well divided by the combined signal of the lane plus the well and multiplied by 100, as before [15].

## Results

### Cell lysis in the presence of RNase A causes chromosomal fragmentation

Intact circular chromosomes and relaxed large plasmids, when released from cells lysed in agarose plugs, do not enter the gel when subjected to pulsed-field gel electrophoresis [23–26]. In order to test if RNA played a role in the chromosomal structure or immobility in PFGE, we embedded ^32^P-labeled wild-type AB1157 cells in agarose plugs in the presence or absence of proteinase K and RNase A, and lysed them by our standard procedure. Without RNase A addition, there is minimal migration of labeled chromosomes from wells into the gel, independently of whether Proteinase K is added or not, indicating no fragmentation (Fig 1B, lanes 1 and 3). Addition of RNase A into plugs, however, caused significant formation of sub-chromosomal fragments in a proteinase K-independent manner, indicating massive chromosome fragmentation (Fig 1B, lanes 2 and 4). We confirmed the signal entering the gel as DNA by treating the lysed plugs with DNase I and observing signal disappearance (Fig 1C).

In order to test whether this chromosomal fragmentation was specific for RNase A, we tested three more RNA degrading enzymes, RNase I_f_, XRN-I and exonuclease T (Exo T). RNase I_f_, like RNase A, is an endoribonuclease [27], whereas XRN-1 is an exoribonuclease requiring RNA ends to start degradation [28]. Exo-T, also called RNase T, is an exonuclease that targets both single-stranded RNA and DNA harboring free 3’ terminus [29,30]. For this assay, we embedded cells in lysis agarose in the presence of various RNases and the appropriate buffer and incubated the solidified plugs in lysis buffer under standard conditions. We found that various RNA-degrading enzymes caused comparable levels of chromosomal fragmentation (Fig 1D and 1E). The minor increase in fragmentation seen in the plugs made with RNase A could be due to higher specific activity of this enzyme. Taken together, data in Fig 1B–1D show that degradation of RNA during the lysis of cells in agarose plugs causes breakage of the chromosome. We call this phenomenon "RNase-induced chromosomal fragmentation" (RiCF).

### RiCF is dynamic in nature

**Fig 2:**
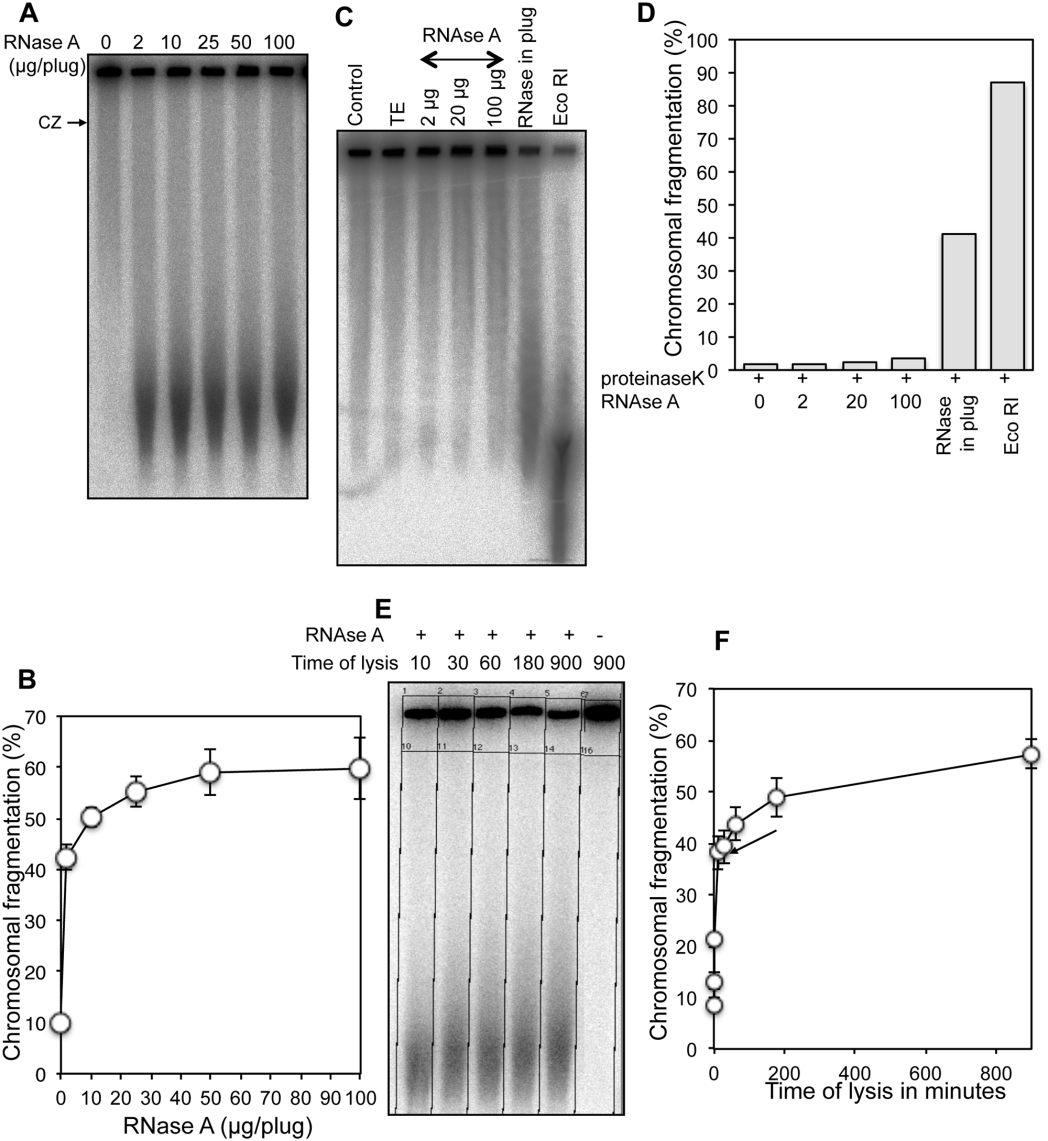
Characterization of RiCF. **(A)** Representative radiogram showing RNase dose dependent chromosomal fragmentation in AB1157. Plugs were made with 0, 2, 10, 25, 50 or 100 μg RNase and lysed and electrophoresed under standard conditions. CZ, compression zone. **(B)** Quantification showing increase in chromosomal fragmentation in RNase dose-dependent manner. Data points are means of six independent assays ± SEM. **(C)** RNase-effect is not seen in the pre-lyzed cells. Plugs from AB1157 culture were made in the presence of proteinase K, but without any RNase. After overnight lysis and extensive washing, the plugs were incubated with 0, 2, 20 and 100 μg RNase or 100 U of EcoRI for 15 H at 37°C before PFGE. **(D)** Quantification of chromosomal fragmentation showing extreme sensitivity of chromosomes to EcoRI, but not RNase, when plugs were treated with the enzymes after lysis of cells. The experiment is done twice and a representative result is presented. **(E)** A representative radiogram showing kinetics of RiCF. Multiple plugs were made in the presence of RNase (50 μg/plug) and incubated at 62°C for 10, 30, 60, 180 or 900 minutes with lysis buffer in individual tubes. At the indicated times, one tube was removed, lysis buffer was replaced with ice-cold TE, and plugs were stored at 4°C until all plugs were ready for electrophoresis. **(F)** Quantification of kinetics of chromosomal fragmentation when plugs were made in the presence of RNase and lysed for 1, 5, 10, 30, 60, 180 or 900 minutes. Data points are means of three independent assays ± SEM. Arrow shows the value of fragmentation after 10 min lysis.

In order to choose the optimal amount of RNase (from here on, we will use only RNase A and will refer to it simply as "RNase"), we performed the dose-dependence of chromosomal fragmentation. As shown in Fig 2A, chromosomes showed extreme susceptibility to the presence of RNase, fragmenting when plugs were made with as little as 2 μg of the enzyme, with a modest dose-dependent increase in fragmentation in the range of 2–50 μg (Fig 2B). Interestingly, while quantity of fragmented DNA increased with the amount of enzyme used, the size of broken DNA fragments stayed the same (Fig 2A). We investigated this observation further (see below).

Based on the result of dose-dependence assay, we used three concentrations of RNase: 2-, 20- and 100 μg,—to determine sensitivity of chromosomal DNA released from cells lysed without RNase. In this assay, cells were embedded in agarose plugs in the absence of RNase, lysed under standard conditions, washed to remove traces of sarkosyl and EDTA, and then the plugs with the released chromosomal DNA were treated with various amounts of RNase. Since the system was "cleaner" in that it lacked any potential RNase inhibiting factors, we expected higher fragmentation under these conditions. To our surprise, however, cells lysed in the absence of RNase failed to exhibit chromosomal fragmentation when subjected to later RNase treatment (Fig 2C and 2D). EcoRI treatment, on the other hand, caused 90% release of the chromosomal DNA from wells (Fig 2C and 2D) confirming its purity and accessibility to enzymes. RNase-resistance of pure chromosomal DNA suggested that, in the process of being released during the cell lysis, nucleoid goes through an RNase-sensitive transition.

Since the standard plug-lysis was done at 62°C for 15H (significant RiCF is observed when the cell lysis is done at physiological temperature of 37°C too), we asked how long it took for RNase to cause chromosome breakage when the enzyme was mixed with cells during agarose embedment. To determine the kinetics of plug lysis and RiCF, we made multiple plugs using same amount of enzyme (50 μg / plug), but lysed them for various periods of time. While plugs made in the absence of RNase and lysed for 15H showed negligible fragmentation (Fig 2E), lysis for only 10 min was sufficient to yield high amount of broken DNA when plugs were made in the presence of RNase, showing that RiCF is fast and efficient. This data also suggested that the bulk of cells were lysed within 10 minutes. Interestingly, similar to the dose-response assay, the smallest size of broken DNA fragments at any time of lysis stayed the same (Fig 2A and 2E). Taken together, we conclude that both the lysis of cells and RiCF in agarose plugs is very fast and requires little RNase activity. We also conclude that RNase needs to be present around the cells during their lysis to cause RiCF.

We further investigated the loss of RiCF in prelysed cells and propose that inactivation of RNase by proteinase K or nucleoid changes during cell lysis could have contributed to it (S1, S2 and S3 Figs). Alternatively, loss of RiCF could be due to the loss of a critical yet uncharacterized factor during cell lysis.

### Growth conditions affect RiCF

**Fig 3:**
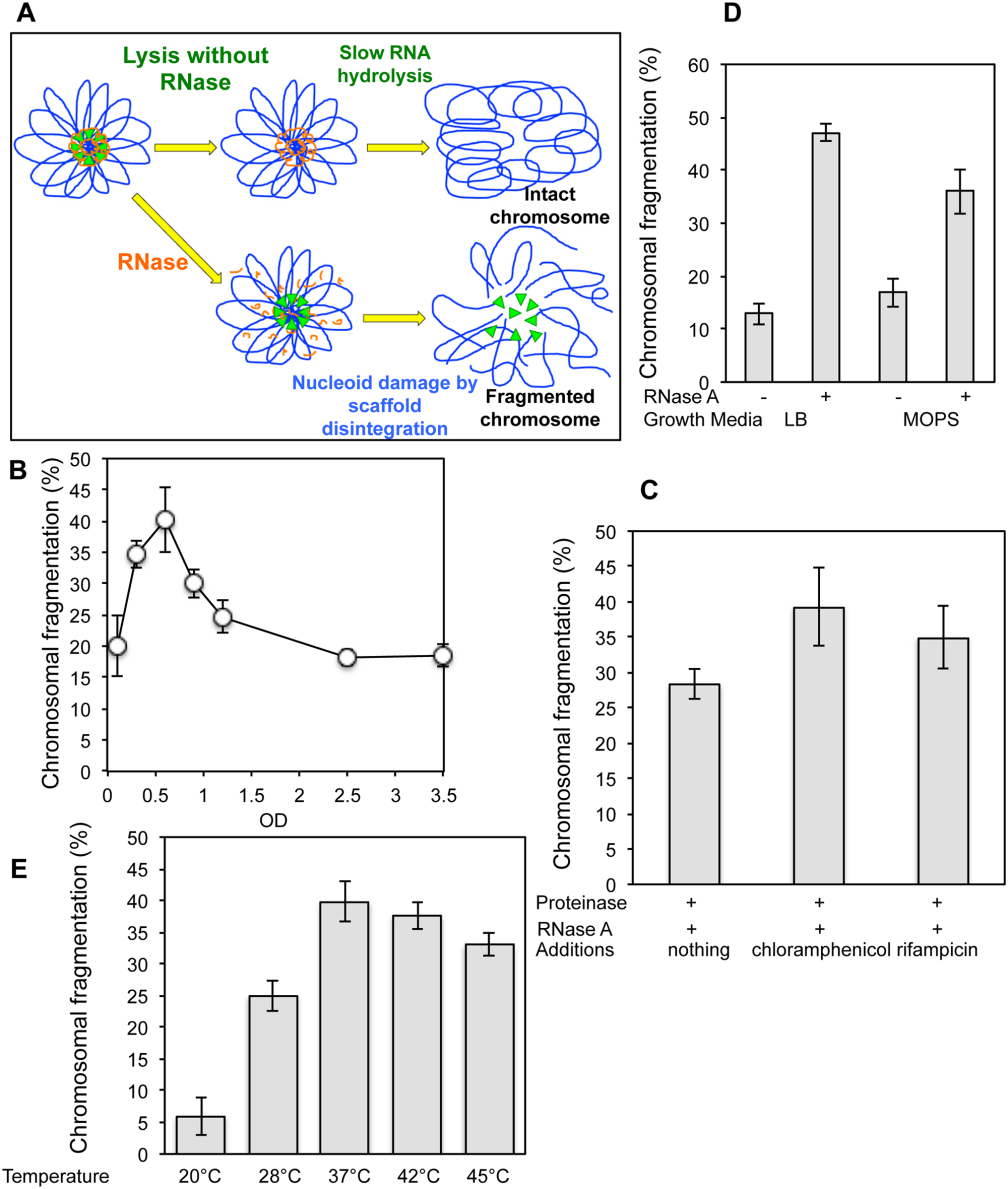
Effect of the growth parameters on RiCF. **(A)** Schematics of a hypothetical scenario when RNA inhibits NAPs that could potentially cleave DNA. During lysis, quick RNA degradation removes the inhibition resulting in breakage of chromosomes. **(B)** Growth phase dependence of RiCF. AB1157 was grown at 37°C with periodic OD measurements, and samples for plugs were withdrawn at various times. The cells were made into plugs using lysis agarose and RNase (50 μg/plug) and the plugs were lysed and electrophoresed under standard conditions. Data points are means of at least three independent assays ± SEM. **(C)** Effect of translation and transcription inhibition on RiCF. Cells were grown till OD 0.5–0.6, split into three parts and chloramphenicol (40 μg/ml) or rifampicin (150 μg/ml) were added to two samples. All sample were shaken for another 2–3 hours at 37°C before making plugs as described in (B). Data points are means of four independent assays ± SEM. **(D)** Growth in minimal medium reduces RiCF. Cells were grown in LB or MOPS till the OD reached 0.6 and made into plugs using standard conditions. The values presented are means of six independent assays ± SEM. **(E)** Effect of growth temperature on RNase-induced chromosomal fragmentation. Cultures of AB1157 were grown at 20°C, 30°C, 37°C, 42°C or 45°C to same cell densities (A_600_ = 0.6), and plugs were made in lysis agarose with RNAse A (50 μg/plug), as described in (A). Data are means of three to six independent measurements ± SEM.

How could degradation of RNA cause such a massive chromosomal fragmentation that it amounted to the disintegration of nucleoids? The simple possibility, that RNA was an integral part of the nucleoid scaffold (Fig 1A), became unlikely because chromosomal DNA of the cells lysed without RNase showed minimal fragmentation and stayed insensitive to subsequent digestion with RNase. Therefore, degradation of RNA caused chromosomal breakage by some other mechanism. The size of the fragmented DNA, which was always in the range of 100–500 kbp (Fig 2A and 2E), suggested that RNA may have participated in the nucleoid stabilization in some periodic manner, for example, inhibiting a potential DNA-breaking nucleoid-associated protein, like a topoisomerase (Fig 3A, discussed in details later). Since nucleoid undergoes structural changes when cells enter the stationary phase [31], we decided to test whether growth parameters influenced RiCF. We chose four such parameters: transition from growth into stationary phase, inhibition of transcription or translation, slowing down of growth rate by use of minimal medium and the growth temperature.

In rapidly growing cultures, RiCF reaches the maximum at OD 0.6 (Fig 3B). Thereafter, as the cells enter the stationary phase, their chromosomes become increasingly resistant to RiCF. In order to test if reduction in RiCF in stationary phase cells was due to decreased transcription or translation, we determined RiCF in cells pre-incubated for two- to three hours in the presence of chloramphenicol (blocks translation) or rifampicin (blocks transcription). Contrary to our expectations, we found that inhibition of translation or transcription didn’t affect RiCF (Fig 3C), suggesting that the presumed "structural nucleoid RNA" is not simply a nascent mRNA [9,32] being generated by transcription elongation complexes, which are known to cluster together and stay attached during nucleoid isolation [33,34].

In order to test if the rate of growth influenced RiCF, we grew AB1157 in LB or in MOPS minimal medium (supplemented with 0.2% casamino acids and 0.2% glucose) and made plugs in the presence or absence of RNase for the fragmentation analysis. As expected, cells grew slowly in MOPS and took longer time to reach OD 0.6. In comparison to LB-grown cells, MOPS-grown cells exhibited slightly higher spontaneous fragmentation (Fig 3D), but reduced RiCF (translating into a significant reduction in the net RiCF). We conclude that fast growth in rich medium promotes RiCF.

The temperature of growth is known to affect DNA supercoiling and therefore the structure of the nucleoid [35]. We found that cells grown at 37°C, 42°C and 45°C exhibited comparably high levels of RiCF. In comparison, there was a mild reduction in RiCF in cells grown at 28°C, and a minimal RiCF in cells grown at 20°C (Fig 3E). Reduction in RiCF at 20°C and 28°C could be due to long-term changes in the cell physiology or due to short-term reaction of the cells to low temperatures. In order to distinguish between these possibilities, we grew the cells at 37°C and chilled them on ice before making plugs. We found no reduction in RiCF between chilled and non-chilled cells confirming that long-term changes in the cell physiology were responsible for reduced RiCF in cells grown at low temperatures.

### Genetic analysis of RiCF

The results so far confirmed that quick lysis of cells in agarose plugs, concomitant with non-specific degradation of RNA, caused breakage of chromosomes. While RNase treatment has been shown to loosen nucleoids [9,10,36], whether specific proteins are also involved in this relaxation is not known. We ruled out the possibility of RNA being an integral part of the nucleoid (as shown in Fig 1A), as cells lysed in the absence of RNase not only became insensitive to RNase treatment, but also showed no increase in spontaneous fragmentation. Another disqualified possibility was fragmentation due to the nucleoid attachment to cell envelope via mRNA bridges, as RiCF was not affected by transcription inhibition [32,37]. Also, chromosomes of cells lysed in the absence of RNase should have shown increased fragmentation, because cell envelopes are destroyed during lysis. Finally, an RNA component could be required for the association of a protein or inhibition of a potential DNA-breaking enzyme involved in the nucleoid structure, and removal of such RNA by RNase treatment could lead to breaks in the chromosomes (Fig 3A). In general, if there is a complex RNA-protein-DNA interaction, interference in which leads to introduction of breaks in DNA, mutations in some nucleoid-associated proteins are expected to exhibit an effect on RiCF.

To identify such proteins, we created strains defective in several NAPs, as well as enzymes involved in RNA metabolism, and measured spontaneous and RNase-induced chromosomal fragmentation in these mutants. We found that single mutations in *hupA*, *hupB*, *ihfA*, *ihfB*, and *recN* didn’t affect the spontaneous fragmentation observed in the absence of RNase, whereas *fis* and *stpA* mutations increased it slightly (S4 Fig). The increase in spontaneous fragmentation in the *fis* mutant was due to the appearance of a low molecular weight DNA species (S4B Fig). Similar, low molecular weight species occasionally appeared in *hup* single and double mutants (see below). None of these mutations, maybe with the exception of *ihfB* single mutant, caused any significant reduction in RiCF (S4 Fig).

**Fig 4:**
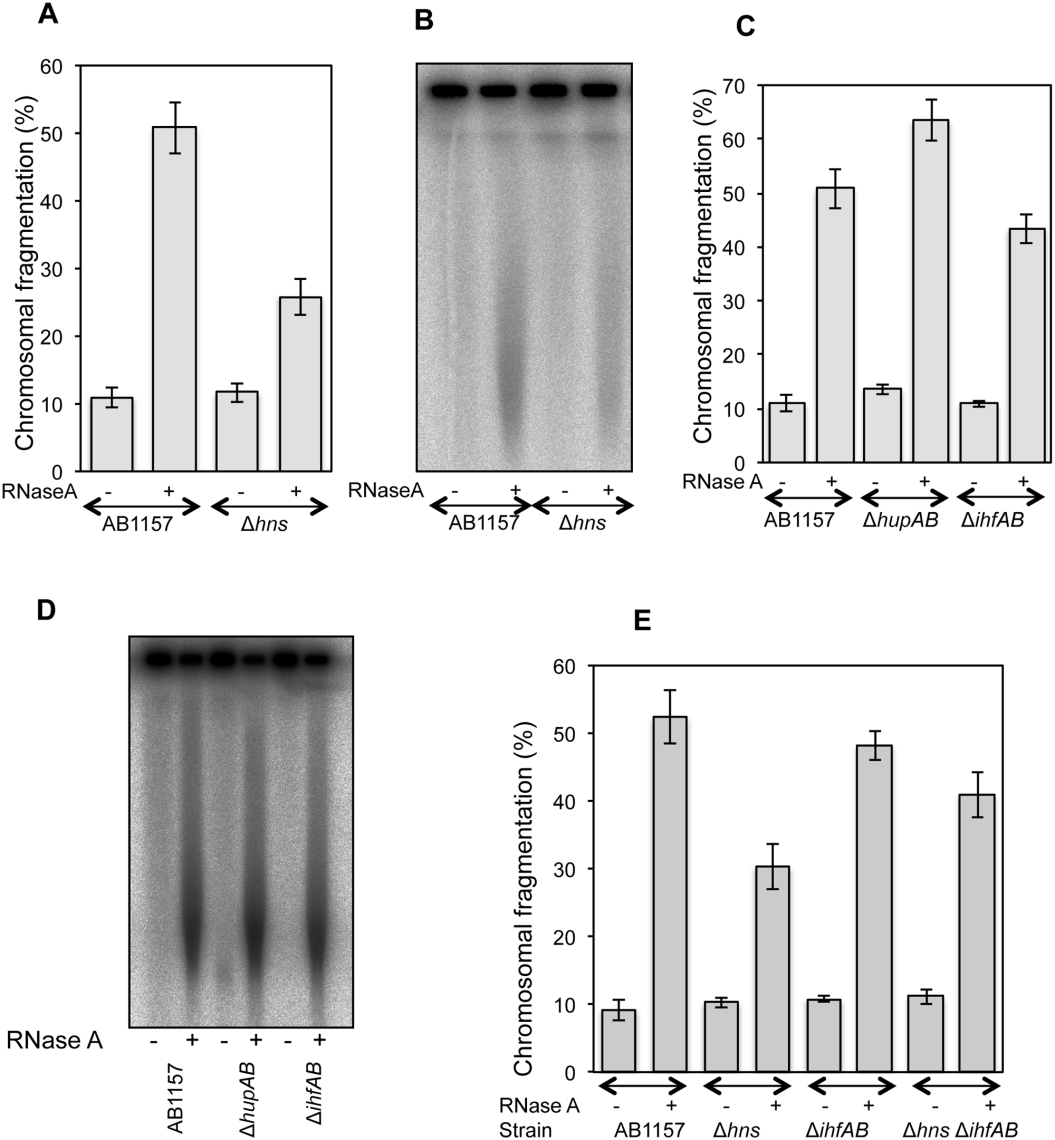
Genetics of RiCF. **(A)** Quantitative determination of RiCF in Δ*hns* mutant. AB1157 and SRK254 were grown at 37°C to the same final OD, and plugs were made in the absence of proteinase K, but with or without RNase (50 μg/plug). After overnight incubation in the lysis buffer at 62°C, the plugs were electrophoresed under standard conditions. Data points are means of 6–10 independent assays± SEM. **(B)** Radiogram of a representative pulsed field gel from which data in (A) are calculated. **(C)** Comparison of RiCF in Δ*hupA* Δ*hupB* and Δ*ihfA* Δ*ihfB* double mutants. Experiment was done as described in (A), and values presented are means of 6–13 independent assays ± SEM. **(D)** Radiogram of a representative pulsed field gel from which data in (C) are calculated. **(E)** Effect of *hns* deletion on RiCF of Δ*ihfA* Δ*ihfB* mutant. Values presented are means of 7 independent assays ± SEM.

Deletion of *hns* gene, however, showed significant reduction in RiCF (Fig 4A and 4B), highlighting the potential role of this protein. Since the NAP single mutants, save one, have normal RiCF, to reveal possible redundancies among NAPs we created *ihfAB* and *hupAB* double mutants and observed RiCF reduction in *ihfAB* mutant, but not in the *hupAB* mutant (Fig 4C and 4D). In fact, *hupAB* double mutant showed modest increase in RiCF (Fig 4C). No further reduction in RiCF was observed in the *ihfAB hns* triple mutant, though (Fig 4E).

We also tested two mutants defective in 3’ to 5’ exoribonucleases, RNase R (*rnr*) and PNPase (*pnp*), and found that neither mutation caused any significant change in the net RiCF. At the same time, deletion of *pnp* gene, which codes for polynucleotide phosphorylase, caused a modest increase in spontaneous fragmentation (S5 Fig). This was puzzling, since PNPase is associated with terminal degradation of RNA fragments [38], and its deletion, causing reduction in the overall RNA turnover, was expected to decrease spontaneous fragmentation. However, loss-of-function mutations in *pnp* gene have also been shown to decrease the stability of several small non-coding RNAs (sRNAs) [39], so the effect on spontaneous fragmentation in the *pnp* mutant could have indirect nature, via destabilizing the protein scaffold of the nucleoid (Fig 3A). From these results we conclude that H-NS and IHF proteins are required for maximal RiCF, while StpA, Fis and PNPase could play minor roles.

### Target RNA and the nucleoid associated proteins

**Fig 5:**
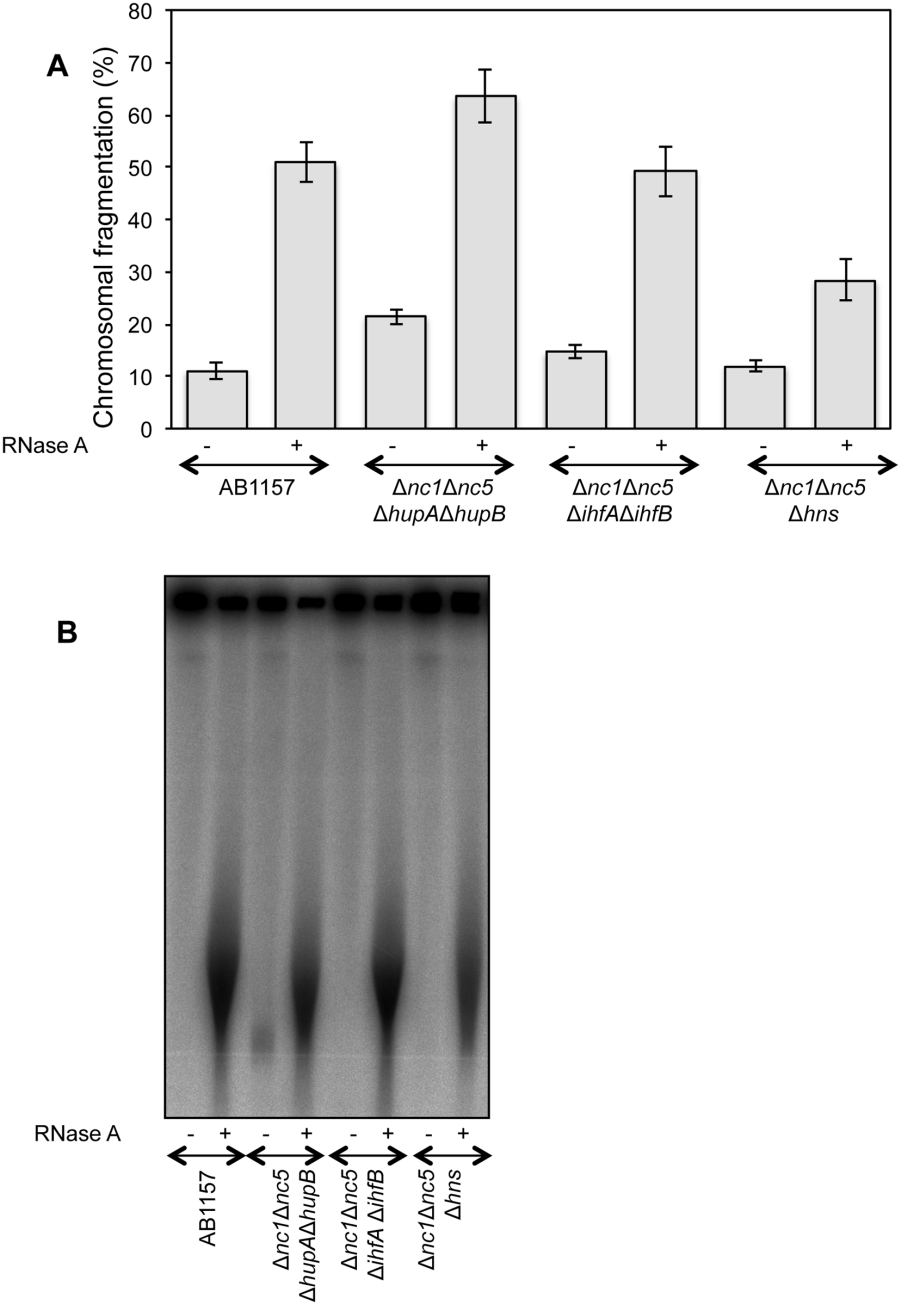
Non-coding RNA and HU stabilize nucleoids. **(A)** Comparison of spontaneous and RNase-induced fragmentation in Δ*nc1* Δ*nc5* Δ*ihfA* Δ*ihfB*, Δ*nc1* Δ*nc5* Δ*hupA* Δ*hupB* and Δ*nc1* Δ*nc5* Δ*hns* mutants. AB1157, SRK254-12, SRK254-15 and SRK254-18 were grown at 37°C, and plugs were made in absence of proteinase K, both with or without RNase (50 μg/plug), and lysed under standard conditions. Data points are means of at least three independent assays± SEM. **(B)** Radiogram of a representative gel from which data in (A) are generated.

Recently, using RNA immunoprecipitation (IP) and microarray profiling (RIP-Chip), Macvanin *et*. *al*. have identified multiple RNA molecules that interacted with HU and described the effect of deletion of two small noncoding RNAs, *nc1* and *nc5*, and heat-unstable nucleoid protein (HU) on nucleoid compaction [12]. In our hands, deletion of *nc1* and *nc5*, individually or in combination, caused no qualitative change in RiCF (the size of fragmented DNA stayed the same), and showed minor reduction when the fragmentation was quantified (S6 Fig). The Δ*nc1* Δ*nc5* double mutant also showed no statistical difference in spontaneous fragmentation but showed, sometimes, low molecular weight DNA. This result suggested that these non-coding RNAs were not the major targets for RNase. The quadruple mutant Δ*nc1* Δ*nc5* Δ*ihfA* Δ*ihfB* also didn’t show any additional difference and behaved like Δ*ihfA* Δ*ihfB* double mutant showing somewhat decreased RiCF (Fig 5). The Δ*nc1* Δ*nc5* Δ*hupA* Δ*hupB* mutant, however, exhibited statistically significant increase in spontaneous fragmentation, compared to both the Δ*nc1* Δ*nc5* and Δ*hupA ΔhupB* double mutants (Figs 5A and 4C and S6 Fig). The spontaneous fragmentation was exclusive to Δ*nc1* Δ*nc5* Δ*hupA* Δ*hupB* quadruple mutant, as Δ*nc1* Δ*nc5* Δ*hns* and Δ*nc1* Δ*nc5* Δ*ifhA* Δ*ifhB* mutants did not show any increase in this species (Fig 5). We conclude that HupAB proteins, especially in combination with the non-coding RNAs *nc1* and *nc5*, contribute to chromosomal stabilization, but *nc1* and *nc5* by themselves are not the targets of RNase for RiCF.

### Is RiCF due to topoisomerases?

One group of nucleoid-associated enzymes that contribute to chromosomal compaction and could potentially break DNA are topoisomerases [40,41]. We tested the possible RiCF-topoisomerase connection by inhibiting DNA gyrase and by growing *E*. *coli* in various concentrations of salt, the conditions that modulate DNA supercoiling.

As shown in S7A Fig, spontaneous fragmentation in *gyrB*(TS) mutant was somewhat increased under non-permissive conditions, causing minor decrease in the net RiCF. The modest effect was confirmed in assays using DNA gyrase inhibitors nalidixic acid, novobiocin and ciprofloxacin (S7B Fig). Ciprofloxacin also showed the expected increase in the background fragmentation, confirming the efficiency of this known clastogen [23,42].

That modulation in DNA supercoiling played no role in RiCF was further confirmed with cells grown in the presence of various concentration of salt, a condition that changes the osmolarity and hence chromosome supercoiling [43–46]. We found that cells grown in LB supplemented with 0-, 0.5- and 1% NaCl exhibited identical RiCF, whereas 2.5% salt caused inhibition in cellular growth and a modest increase in RiCF (S8 Fig). Taken together, the results in S7 and S8 Figs show that interference with DNA topoisomerases or modulation of supercoiling do not significantly affect RiCF.

### RiCF is mediated by endonuclease I

**Fig 6:**
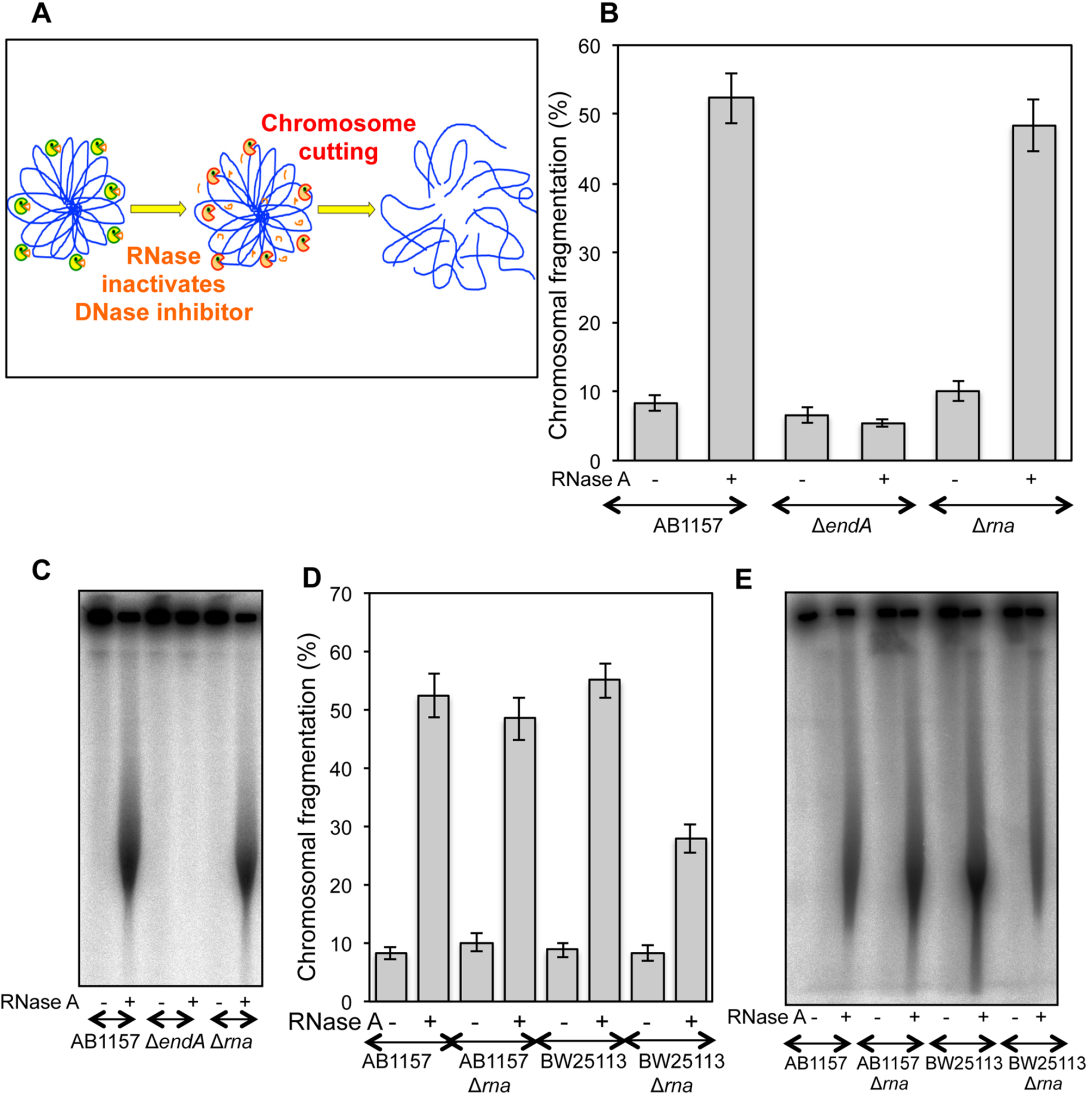
Endonuclease I mediated RNA degradation causes RiCF. **(A)** Schematics of a hypothetical scenario when RNase activates a DNase by degrading an inhibitory RNA. Activated DNase, in turn, attacks chromosomal DNA causing its fragmentation. **(B)** Quantitative determination of RiCF in Δ*endA* and Δ*rnaA* mutants. The strains were grown at 37°C to the same final OD, and plugs were made in the absence or presence of RNase (50 μg/plug). Data points are means of 4–8 independent assays ± SEM. **(C)** Radiogram of a representative pulsed field gel from which data in (B) are calculated. **(D)** Comparison of RiCF in Δ*rna* mutants in two genetic backgrounds. AB1157, BW25113 and their Δ*rna* mutants were grown and processed as described in (A). Values presented are means of 3–8 independent assays ± SEM. **(E)** Radiogram of a representative pulsed field gel from which data in (D) are derived.

So far our results (Fig 3 and S4, S5, S6, S7 and S8 Figs) showed RiCF insensitivity to most mutations in NAPs, factors affecting DNA supercoiling, as well as compaction and membrane attachment of nucleoids [32,37], strongly suggesting that, contrary to our initial expectation, the target RNA may not be associated with the nucleoid. We, therefore, searched for *any* enzyme, not necessarily associated with the nucleoid, that would be 1) inhibited by RNA; 2) could break DNA if the inhibitor RNA is removed (Fig 6A). One such RNA-regulated DNase is endonuclease I, a potent cell envelope-associated deoxyribonuclease of unclear biological function. Endonuclease I is inhibited by a variety of RNAs including t-RNA and r-RNA *in vitro* [47–48], and is found in an RNA-bound state in crude extracts [47], suggesting that the enzyme may stay inactive *in vivo*. Interestingly, the enzyme is believed to be located in the periplasm of the *E*. *coli* cells, separated from the chromosomal DNA by the cytoplasmic membrane [49–51].

In order to determine whether endonuclease I contributed to RiCF, we tested an *endA* deletion strain in two genetic backgrounds, AB1157 and BW25113. As shown in Fig 6B and 6C, and unlike any of the results we have seen before, *endA* mutants were completely devoid of any RiCF in both AB1157 and BW25113 backgrounds (Fig 6, data for BW25113 were indistinguishable). Interestingly, *endA* mutant showed no statistically significant decrease in spontaneous fragmentation, indicating that the enzyme stays inactive under normal plug lysis conditions, possibly because of inhibitory RNA. Apparently, this inhibitory RNA was degraded when RNase was added during plug preparation, and the activated endonuclease I attacked chromosomal DNA during lysis of cells in agarose plugs.

Because it is released during osmotic shock or spheroplast preparation, endonuclease I has been proposed to localize in the periplasmic space [50,51]. Interestingly, the same conditions also released ribonuclease I, a nonspecific endoribonuclease similar to RNase A [49,51–55]. In order to determine if ribonuclease I contributed to RiCF, we tested chromosomal fragmentation in the *rna* deletion strain, again using AB1157 and BW25113 backgrounds. As shown in Fig 6B and 6C, *rna* deletion did not affect RiCF in the AB1157 background. On the other hand, BW25113 *rna* mutant showed significant reduction in RiCF (Fig 6D and 6E) suggesting that RiCF in this background is contributed by both externally added RNase and endogenous ribonuclease I. Since spontaneous fragmentation in both Δ*endA* and Δ*rnaA* mutant stays low, it was conceivable that RNase A initially activated both enzymes, while the activated ribonuclease I then activated endonuclease I. In order to determine whether RiCF can be completely eliminated in the *rna* mutant by reducing RNase, we tested RiCF in WT and *rna* mutant by making plugs using either low (2 μg) or regular (50 μg) amounts of RNase. The results show further decrease, but not elimination, of RiCF with low RNase (S9 Fig). Furthermore, we tested whether reduced RiCF in Δ*hns* mutant was dependent on ribonuclease I and found no further reduction in RiCF of Δ*hns* Δ*rna* double mutant. We conclude that the majority of RiCF is achieved by direct activation of endonuclease I by RNase A.

### Nucleoid anomalies can break the chromosomes

Complete dependence of RiCF on endonuclease I strongly suggested that the target RNA was not associated with nucleoids. Since we also discovered that mutations in *fis*, *pnp*, *hupA hupB* and *nc1 nc5 hupA hupB* caused spontaneous chromosomal breakage, we asked whether endonuclease I could be responsible for this enhanced spontaneous fragmentation. The mild increase in spontaneous fragmentation in Δ*pnp* mutant (S5 Fig, check the fragmented DNA in S5B Fig) was partially dependent upon endonuclease I (S10 Fig). Interestingly, the low molecular weight fragmented species of Δ*pnp* persisted in Δ*pnp* Δ*endA* double mutant indicating a possible role of Pnp in the nucleoid maintenance (S10 Fig).

**Fig 7:**
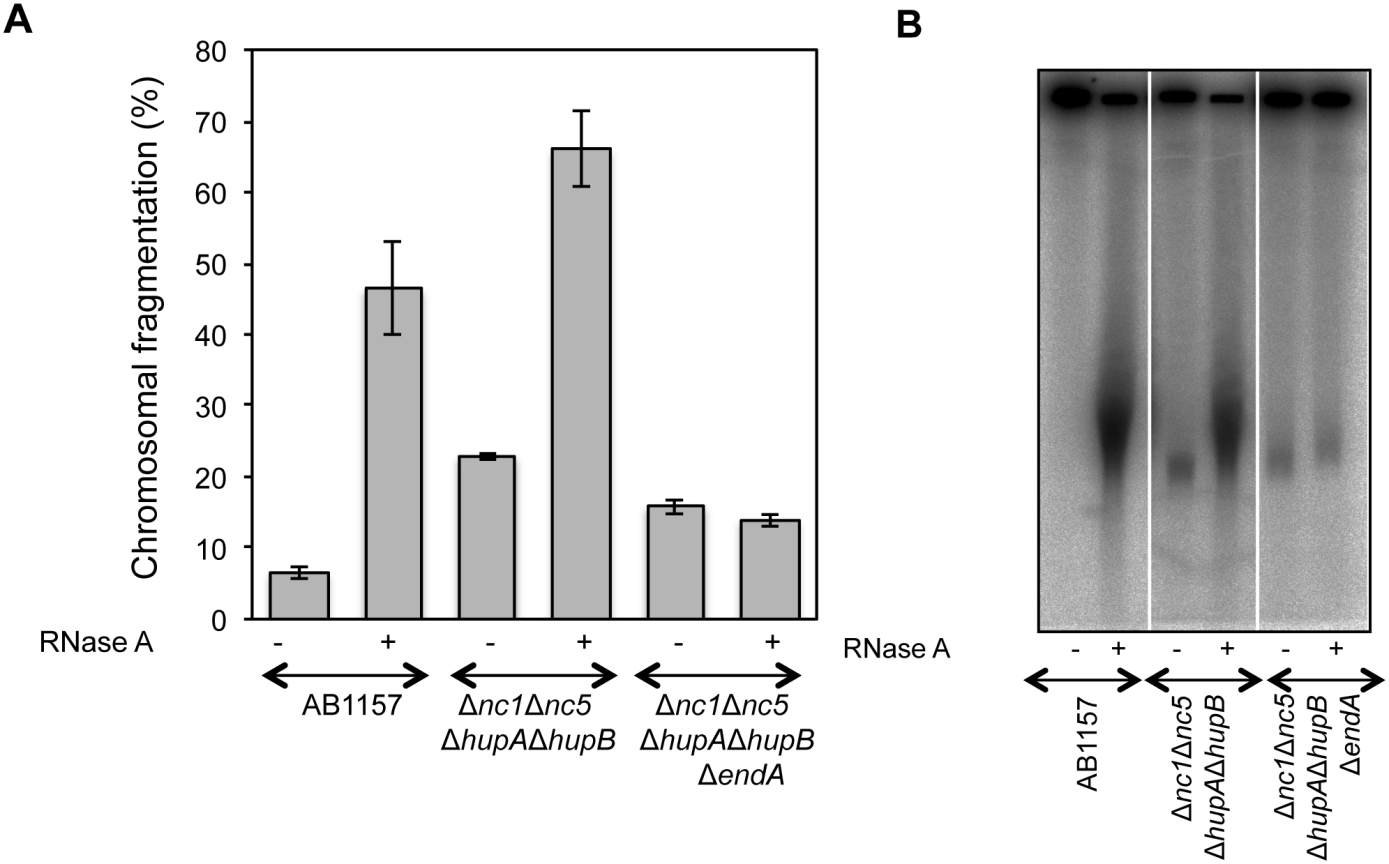
Endonuclease-I is critical for RiCF but not for spontaneous fragmentation. **(A)** Comparison of spontaneous fragmentation and RiCF in AB1157, AB1157 Δ*nc1* Δ*nc5* Δ*hupA* Δ*hupB* and AB1157 Δ*nc1* Δ*nc5* Δ*hupA* Δ*hupB* Δ*endA* mutants. All strains were grown at 37°C to the final OD of 0.6, and plugs were made in the absence of proteinase K, both with or without RNase (50 μg/plug). Data points are means of at least three independent assays ± SEM. **(B)** Radiogram of a representative gel from which data in (A) are generated.

Likewise, endonuclease I was not critical for the robust spontaneous fragmentation in the Δ*nc1* Δ*nc5* Δ*hupA* Δ*hupB* quadruple mutant (Fig 7A). In order to investigate whether spontaneous fragmentation accumulated in a growth phase dependent manner, we compared fragmentation between EndA+ and EndA- derivatives of Δ*nc1* Δ*nc5* Δ*hupA* Δ*hupB* after growing them to various optical densities. As shown in S11 Fig, the spontaneous fragmentation in quintuple mutant stays largely unchanged during growth but shows increase in quadruple mutant, similar to growth phase-dependent RiCF (Fig 3A). These data are in agreement with the results of Macvanin et. al., who reported structural problems in the nucleoid of Δ*nc1* Δ*nc5* Δ*hupA* Δ*hupB* quadruple mutant [12]. We would like to emphasize at this point that, while the strain shows growth defect, we have not tested whether chromosomal breaks in quadruple mutant are generated during cellular growth or during cell lysis.

### Exogenous RNase is not critical for endonuclease I activation

The physiological role of endonuclease I is unclear in *E*. *coli*. It is also not known whether the enzyme exists in the inactive form in the periplasm or gets inactivated during cell lysis due to the release of inhibitory RNA from the cytoplasm [47]. In our assays, RNase activates endonuclease I by degrading either the inhibitory periplasmic RNA (if the enzyme existed in the inactive form in the periplasm), or the RNA released from the cytoplasm (if the enzyme existed in the active form in the periplasm). Our normal lysis conditions in which both plug agarose and lysis buffer contain EDTA and sarkosyl yield low spontaneous fragmentation, confirming that cell lysis in agarose plugs doesn’t lead to endonuclease I activation. Interestingly, we also found that increasing EDTA in the plugs decreases RiCF (S3 Fig) but does not influence the spontaneous fragmentation, suggesting that incomplete metal ion chelation was not the main reason for spontaneous fragmentation. Therefore, if endonuclease I existed in the inactive form in the periplasm, its activation required both removal of inhibitory RNA and the presence of metal ions. Alternatively, if endonuclease I existed in the non-RNA bound form in the periplasm, its activation required only the metal ions. In order to distinguish between these possibilities, we removed EDTA from both plug agarose and lysis buffer (the cells were still suspended in TE) and determined spontaneous fragmentation and RiCF. Under this condition, the amount of EDTA in plugs is 0.5 mM (which are lysed in lysis buffer without any EDTA) as opposed to regular lysis condition in which the amount of EDTA in plugs is 3 mM (which are lysed in lysis buffer containing 25 mM EDTA). As shown in S12 Fig, removal of EDTA from the lysis buffer resulted in massive chromosomal fragmentation (S12 Fig). This fragmentation was partially dependent upon endonuclease I, suggesting that EDTA keeps in check endonuclease I as well as other cellular DNases during regular cell lysis. That half of the fragmentation observed in EDTA-less lysis buffer condition was still dependent upon endonuclease I suggests that the enzyme stayed active during lysis and therefore existed in the non-RNA bound active state in vivo (S12 Fig). This result suggests that under EDTA^-^ lysis buffer conditions, active endonuclease I (together with other nucleases) breaks the chromosomes while simultaneously getting inactivated by the cytoplasmic RNA. This idea is supported by the fact that addition of RNase fragmented the chromosomes even further, but only in endonuclease I^+^ cells, causing the smaller fragments to run out of the gels. Our standard quantification of chromosomal fragmentation procedure severely underestimates this massive fragmentation, because of this loss of signal (S12 Fig, compare signal in wells). We conclude that at least a fraction of endonuclease I exists in the non-RNA-bound state in the periplasm, but is quickly inactivated by the cytoplasmic RNA in the event of cytoplasmic membrane rupture. To sum up, endonuclease I requires at least two factors to fragment the chromosomes efficiently: the presence of an EDTA-chelatable ion and the absence of inhibitory RNA. The spontaneous fragmentation in our standard lysis conditions (EDTA^+^, RNAse^-^) stays low and endonuclease I-independent (S6 Fig) because of strong inhibition of endonuclease I by RNA, rather than due to chelation of *all* metal ions. Under our lysis conditions, metal ions, while presumably still present in small amounts, are not enough to cause spontaneous chromosomal breakage. The importance of the inhibition of the enzyme by RNA is further highlighted by the fact that 5x higher concentration of EDTA fails to reduce spontaneous fragmentation (S3 Fig), but significantly reduces RiCF. This suggests that, while some metal ions are required for robust RiCF, presence or inhibitory RNA can still inactivate endonuclease I.

## Discussion

In this study we report a novel phenomenon, RNase-induced chromosomal fragmentation (RiCF), in which chromosomal DNA of *E*. *coli* is massively fragmented during lysis in agarose plugs in the presence of RNases. This phenomenon exhibited a size-range limit of broken chromosomal fragments, initially suggesting a nucleoid-organization role of degraded RNA, either by itself, or in association with NAPs (Figs 1A and 3A). While RNA was always suspected to stabilize the chromosomal structure [56], and purified nucleoids have been shown to lose compactness upon RNase treatment [9,10,36], we show, for the first time to our knowledge, that the presence of exogenous RNase during cell lysis in agarose plugs caused chromosomal breakage. However, we also show that chromosomes released in agarose plugs without RNase do not fragment and are resistant to subsequent RNase treatment, making the structural role of RNA unlikely.

Out of three main factors that influence nucleoids: DNA supercoiling, inhibition of transcription and NAPs,—only deletion of H-NS, a protein that preferentially binds to curved DNA [57] and is critical for creating domain boundaries [58], reduced RiCF. H-NS is a known regulator of many genes, controlling multiple functions [59–61], and has also been shown to alter the stability of RNA, including noncoding RNA (ncRNA) DsrA [62]. DsrA was not only more susceptible to cleavage by RNase I *in vitro* upon H-NS binding, but was degraded more rapidly *in vivo* in an H-NS-dependent manner [62]. Therefore, it was conceivable that during RiCF, RNase targeted a ncRNA in an H-NS-dependent manner. Reduction of RiCF in Δ*hns* mutant could be due to a decreased production, binding, or both, of the target RNA, or could be due to changes in the degradation of target RNA. We would like to state that, since the *E*. *coli* Δ*hns* mutant exhibits slow growth, defective motility and mucoidy, and is sensitive to high osmolarity, among other anomalies [61], our results do not rule out the possibility of a mechanism that involves a non-RNA factor (see below).

While H-NS was unique in affecting RiCF, *endA*-independent spontaneous fragmentation in certain mutants suggested general nucleoid problems (Fig 7 and S5 Fig). The nucleoid anomalies of Δ*nc1* Δ*nc5* Δ*hupA* Δ*hupB* mutant are in agreement with the report of Macvanin *et*. *al*. who showed that nucleoids lose their characteristic structure in strains harboring these mutations [12]. While not a traditional NAP, in a recent study Pnp has been co-purified with the nucleoid fractions of *E*. *coli*, *Bacillus subtilis*, *Pseudomonas aeruginosa* and *Streptococcus aureus* [5]. While in that study Pnp was not designated as a ‘contaminant-subtracted NAP’ (csNAP), our data suggest that it could have a role in the nucleoid integrity. In both mutants, enhanced spontaneous fragmentation could be due to nicks or breaks in the chromosomes, and at present we do not know whether the chromosomal breaks are the cause or consequence of the nucleoid dispersal during growth, or are generated during cell lysis.

**Fig 8:**
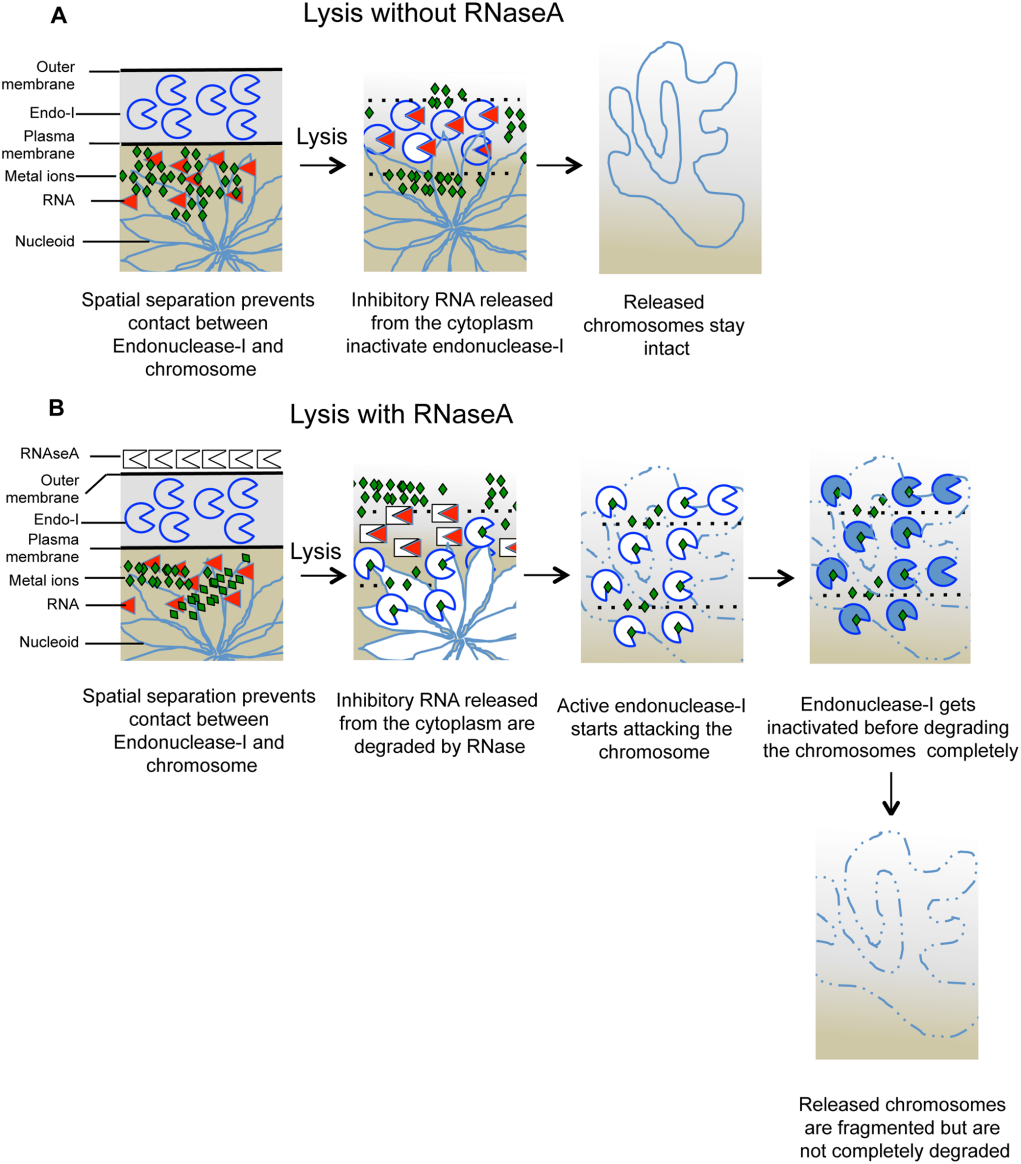
The model. RiCF is generated by activation of endonuclease I. When cells are embedded in agarose plugs without RNase (scenario A), disintegration of plasma membrane during lysis causes release of cytoplasmic RNA that inactivates periplasmic endonuclease I and saves the chromosomes from degradation. RNAse, when present in close proximity of the lysing cells (scenario B), degrades the released cytoplasmic RNA avoiding endonuclease I inhibition. Endonuclease I, in the presence of divalent cations, cuts the chromosomal DNA, but fails to degrade the chromosomes completely because of its prompt inactivation in lysis conditions.

Our discovery that the RNase-dependent fragmentation of the chromosome during lysis was through activation of endonuclease I explained the growth phase-dependence of RiCF (Fig 3B), as the activity of the enzyme in the growing cultures of *E*. *coli* is known to go down sharply as the cells enter the stationary phase [63]. Endonuclease I is a potent nuclease [47,48,64,65] and, in *E*. *coli*, its activity is supposedly kept in check in two ways: by compartmentalization in the periplasmic space and by inhibition with non-specific RNAs [47,50,51,66]. Activation of the enzyme is detrimental for cellular DNA, as McIntosh and Earhart have shown that, during T4 infection, treatment of cell lysates with ribonuclease degraded both host and phage DNA from Mg^2+^-sarkosyl crystals (M-bands) [67]. RiCF conditions compromise both the compartmentalization and the RNA inhibition checks on the activity of endonuclease I, putting the active nuclease in direct contact with susceptible chromosomal DNA (Fig 8 and S13 Fig).

Two pieces of evidence suggest that activation of endonuclease I is not limited to RNase treatment. First, removal of EDTA from plug agarose and lysis buffer caused spontaneous fragmentation which was partly dependent upon endonuclease I (S12 Fig), in agreement with the *in vitro* observation that removal of Mg^2+^ ions reduced Endo I activity, while addition of 25 mM EDTA caused further inhibition [68]. Second, our preliminary results show that plugs made from osmotically-shocked cells, when incubated in the presence of DNase I buffer, also fragmented their chromosomes, but only when the cells were endonuclease I-proficient. As a positive control, both WT and *endA* mutant fragmented their chromosomes when osmotically shocked cells were incubated with DNase. Both lines of evidence support the idea that at least part of the endonuclease I may exist in a non RNA-bound state in the periplasm (Fig 8 and S13 Fig).

Finally, if RiCF is completely dependent upon endonuclease I, how does H-NS mutant show reduction in fragmentation in endonuclease I^+^ strain? We first suspected slow growth and mucoidy of the H-NS mutant to be the culprit, but found that Δ*ihfAB hns* triple mutant retained these characteristics, yet showing improved RiCF (Fig 4E). The *endA* gene was also not regulated by H-NS [59]. One possible explanation behind reduced RiCF in Δ*hns* mutant could be that H-NS modulates the amount of inhibitory RNA, causing poor activation of endonuclease I. Alternatively, decreased RiCF in H-NS^-^, endonuclease^+^ cells could be due to an altered nucleoid structure which becomes a poor substrate for endonuclease I [47] or is not readily released from cells during lysis.

Why did the activated endonuclease I fail to degrade the entire chromosome? Based on the results in this study, we propose that RiCF is a multifactorial process and could be influenced by metal ions chelation, structural changes of the nucleoid, or enzyme denaturation during lysis (Fig 8). Which of these factors limit the ability of enzyme to degraded chromosomes completely remains to be determined. A physical parameter, restriction of movement of macromolecules imposed by embedment of lysing cells in the agarose plugs, together with the inactivation of endonuclease 1, could also stop complete degradation of chromosomes (S13 Fig).

In conclusion, our study shows, for the first time to our knowledge, that RNA degradation during *E*. *coli* cell lysis in agarose plugs causes fragmentation of the chromosomes, by apparently activating endonuclease I at the time of highest DNA vulnerability. Our study also gives indirect evidence that endonuclease I may exist in non-RNA bound state in *E*. *coli* and suggests that the cell may specifically want to inhibit endonuclease I with the cytoplasmic RNAs, so that the enzyme does not attack chromosomal DNA in case the cytoplasmic membrane is transiently ruptured. Such scenario offers a biological meaning of our finding in that Gram-negative cells can safely keep a powerful non-specific DNase in the periplasm by making the DNase acutely sensitive to inhibition by any cellular RNA molecules (we do not rule out the possibility that a specific RNA is involved). As a result, if the periplasm gets in contact with the cytoplasm, Endo I is instantly inactivated by the flood of cellular RNA, which prevents cleavage of the chromosomal DNA. We speculate that the role of the periplasmic RNase I could be to slowly remove the RNA inhibition from Endo I, once the periplasm/cytoplasm barrier is restored.

As a last note, is RiCF linked to the RNase-induced decompaction of purified nucleoids [9, 10, 36]? We, while not testing it directly, feel the scenario unlikely because during rupture of cell membrane endonuclease I is subject to both inactivation and leaching out, making expansion of purified nucleoids upon RNase treatment unrelated to DNA cleavage by endonuclease I. We suspect that during the lysis of the cells in agarose plugs, RNase by itself either fails to decompact the nucleoids or any RNase-induced change in the compactness of the nucleoids does not translate into generation of double strand breaks. An alternative possibility, that decompaction of the nucleoids by RNase was required for the endonuclease-I dependent RiCF, also exists but has not been tested.

## Supporting information

S1 Fig Conditions of lysis affect RiCF. (PDF)

S2 Fig Effect of proteinase K on RNase dose-dependent fragmentation. (PDF)

S3 Fig Effect of sarkosyl and EDTA in plugs on RiCF. (PDF)

S4 Fig Effect of NAP mutants on RiCF. (PDF)

S5 Fig Effect of deletion of RNA degrading enzymes PNPase and RNase R on RiCF. (PDF)

S6 Fig Effect of deletion of non-coding RNAs *nc1* and *nc5* on RiCF. (PDF)

S7 Fig Effect of inhibition of DNA gyrase on RiCF. (PDF)

S8 Fig Effect of osmolarity of growth medium on RiCF. (PDF)

S9 Fig Effect of deletion of ribonuclease I on RiCF. (PDF)

S10 Fig Effect of endonuclease I on RiCF of Δ*pnp* and Δ*rnr* mutants. (PDF)

S11 Fig Kinetics of spontaneous fragmentation. (PDF)

S12 Fig Plug lysis in EDTA-less lysis buffer causes spontaneous fragmentation. (PDF)

S13 Fig Incomplete degradation of chromosomes during RiCF could be due to contact limitation. (PDF)

## Abbreviations

CZ: compression zone
NAP: nucleoid associated protein
PFGE: pulsed field gel electrophoresis
RiCF: RNase-induced chromosomal fragmentation
