# Supplementary material for: Degradation of RNA during lysis of Escherichia coli cells in agarose plugs breaks the chromosome
Source: PLoS One. 2017 Dec 21;12(12):e0190177. doi: 10.1371/journal.pone.0190177 (PMC5739488; doi:10.1371/journal.pone.0190177)
Supplement: S1 Fig — (PDF) [file pone.0190177.s001.pdf]

Degradation of RNA during lysis of *Escherichia coli* cells in agarose plugs breaks the chromosome

Sharik R. Khan* and Andrei Kuzminov

Supporting information and results

Fast cell lysis with limited EDTA is required for RiCF

The fact that RiCF was very fast when enzyme was added directly to the plug mixture, but no effect was observed when RNase was added after the cells were lysed, strongly suggested the existence of a critical period, during which RNase has to be present around lysing cells for efficient RiCF. In order to test this idea, we made plugs without RNase and incubated them in the lysis buffer containing increasing amounts of RNase followed by the standard lysis for 15 hours. We observed poor chromosomal fragmentation when RNase-less plugs were incubated in lysis buffer containing as high as 1 mg/ml RNase (Figs 3A and B), whereas plugs made with RNase (50 μg/plug) yielded characteristic high fragmentation. Interestingly, unlike previous results when the fragmentation pattern remained low molecular weight with different amounts of RNase or time of time of lysis, the molecular weight of fragmented DNA when RNase was added in the lysis buffer was relatively high (S1 Fig).

The relative insensitivity of the chromosome to RNase added in lysis buffer could mean that either presence of RNase around lysing cells was critical, or that RNase was rapidly inactivated by some factors during the lysis. One likely RNase-inactivating factor was proteinase K. While the presence of proteinase K failed to abolish RiCF under regular conditions (S1B Fig) or caused fragmentation when the plugs were made in the

absence of proteinase K and incubated in lysis buffer containing increasing amounts of RNase (S1C Fig), we directly tested, in two different ways, the possibility that proteinase K could inactivate the RNase. First, we mixed the two enzymes, incubated the mixture at 55°C or 37°C for 5 min, and added this mixture to cells before making agarose plugs. Second, we performed a complete dose response assay to test quantitatively if RNase-effect was susceptible to the presence of proteinase K. Another component to potentially inactivate RNase was the lysis buffer itself which contained sarkosyl and EDTA. If RNase was indeed getting inactivated by the components of the lysis buffer, making plugs in plain agarose (Sarkosyl$^-$, EDTA$^-$) containing RNase, and lysing them regularly in lysis buffer (sarkosyl$^+$, EDTA$^+$), should increase fragmentation.

We found that RiCF was dramatically reduced when plugs were made in agarose lacking sarkosyl and EDTA (S1D and S1E Figs). We also found that, as expected, pre-incubation of RNase with proteinase K significantly reduced the chromosomal fragmentation when the mixture of two enzymes was incubated at 55°C (S1D and S1E Figs), confirming that proteinase K could degrade RNase. However, such degradation was apparently too slow to completely block the chromosomal fragmentation under standard conditions when the two enzymes were added together in plugs (Fig 1B and S1D Fig). Similarly, when included in an RNase dose response assay, proteinase K reduced, but could not abolish, RiCF even at the lowest RNase concentration (S2 Fig).

We propose two explanations for the inability of proteinase K to abolish RiCF in plugs when the two enzymes were added together. First, the lysis and chromosomal fragmentation could be faster than proteinase K induced inactivation of RNase (assuming that cell lysis is required for RNase to gain access to nucleoids). Second, proteinase K

may not be as effective in degrading RNase in agarose plugs as in the solution. The first idea is supported by our observations that very small amounts of RNase could cause chromosomal breakage (even if the majority of it can be degraded by proteinase K) and that making plugs in plain agarose dramatically decreased the fragmentation (Figs 3C and D, see below) suggesting that small amounts of sarkosyl and EDTA (final concentration in plugs 0.1% and 2.5 mM, respectively), which likely ensure faster lysis, were critical for RiCF. We propose that, while cells in plain agarose plugs could be lysed to completion upon incubation in lysis buffer, this process is slow, and therefore RiCF is lost due to either degradation of RNase by proteinase K before cell lysis or loss of another critical activity required for RiCF.

We tested and confirmed that the presence of sarkosyl and EDTA in plugs by themselves were insufficient to lyse the cells if plugs were incubated in TE instead of lysis buffer. Furthermore, when tested individually, both sarkosyl and EDTA were required to recapitulate the RiCF (S3 Fig), suggesting that small quantities of these components in plugs either contributed to faster lysis or facilitated RiCF by affecting some other factor. We also found that increasing the amount of EDTA in plugs from 2.5 mM to 12.5 mM was detrimental and reduced RiCF (S3 Fig). We conclude that both the fast lysis ensured by sarkosyl and small concentration of EDTA in plugs, and the presence of RNase around lysing cells, are required for maximal RiCF. We propose that either inactivation of RNase by proteinase K, or nucleoid changes during cell lysis, or loss of another critical factor, when cells were lysed in the absence of RNase, suppress RiCF when the enzyme is added after lysis of the cells.

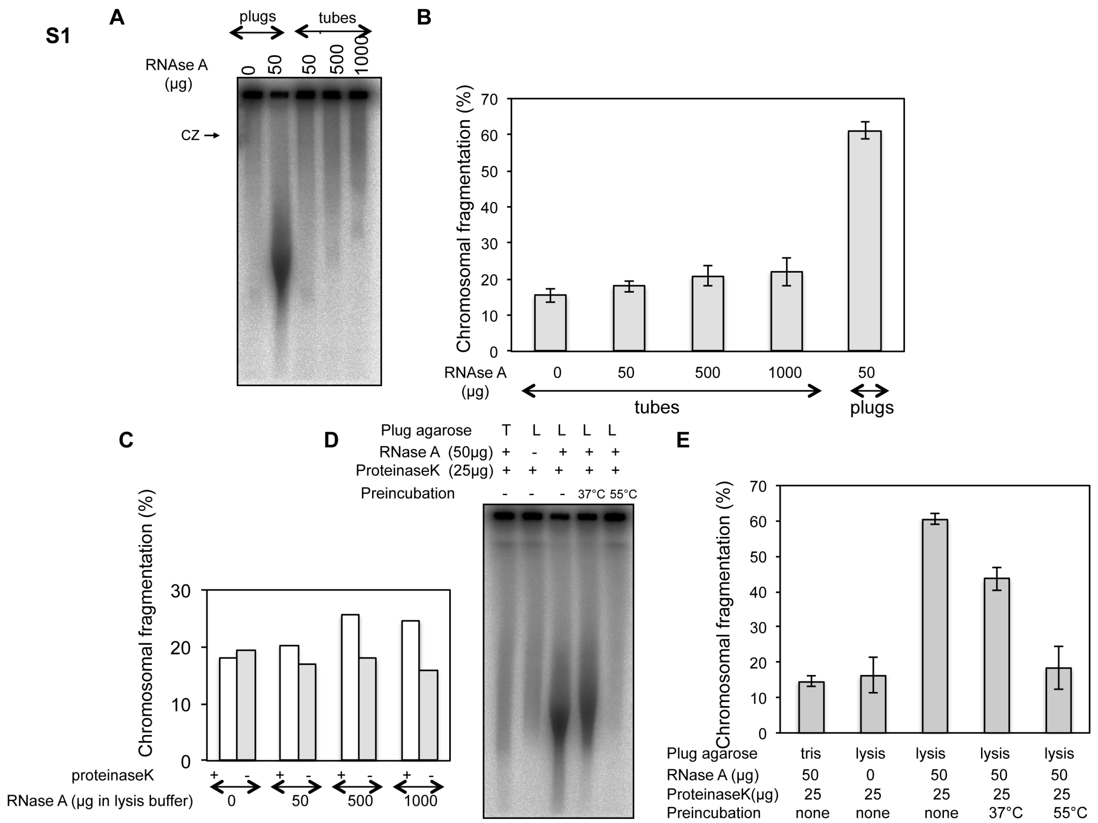

S1 Fig. Conditions of lysis affect RiCF. (A) A radiogram showing fragmentation profile of AB1157 when plugs were made in regular lysis agarose with proteinase K (25 µg/plug) alone. These plugs were lysed under standard conditions in lysis buffer containing 0, 50, 500 or 1000 µg/ml RNase. As a control, one plug was made in the presence of proteinase K (25 µg/plug) and RNase (50 µg/plug) and lysed overnight in lysis buffer at 62°C. CZ, compression zone. **(B)** Quantification of fragmentation showing poor RNase-effect when the enzyme was added in the lysis buffer and not inside plugs. Data points are means of three independent assays ± SEM. (C) Proteinase K in plugs doesn't affect RiCF when RNase was included in lysis buffer. AB1157 was grown until it reached the OD=0.6 and made into multiple plugs without RNase but with either 0 or 25 µg/plug proteinase K. The solidified plugs were transferred into tubes containing lysis buffer with 0, 50, 500 or 1000 µg RNase. The plug lysis and electrophoresis was performed using standard conditions. **(D)** A radiogram showing that sarkosyl and EDTA in plugs are required for RiCF. Cells were embedded in agarose plugs in presence of proteinase K (25 µg/plug) but with or without RNase (50 µg/plug) using either lysis agarose ($EDTA^+$, $sarkosyl^+$) or tris agarose ($EDTA^-$, $sarkosyl^-$). In one plug, RNase and proteinase K were not added individually. Instead, 5 µl of proteinase K (5 mg/ml) and 25 µl of RNase (2 mg/plug) were mixed and incubated at 37°C or 55°C for 5 minute and this mixture was used to make plugs using lysis agarose ($EDTA^+$, $sarkosyl^+$). All plugs were incubated in lysis buffer under standard conditions. **(E)** Quantification of the radiogram

shown in (D). The values presented are means of three to four independent assays ± SEM.
