# Supplementary material for: Degradation of RNA during lysis of Escherichia coli cells in agarose plugs breaks the chromosome
Source: PLoS One. 2017 Dec 21;12(12):e0190177. doi: 10.1371/journal.pone.0190177 (PMC5739488; doi:10.1371/journal.pone.0190177)
Supplement: S2 Fig — (PDF) [file pone.0190177.s002.pdf]

S2

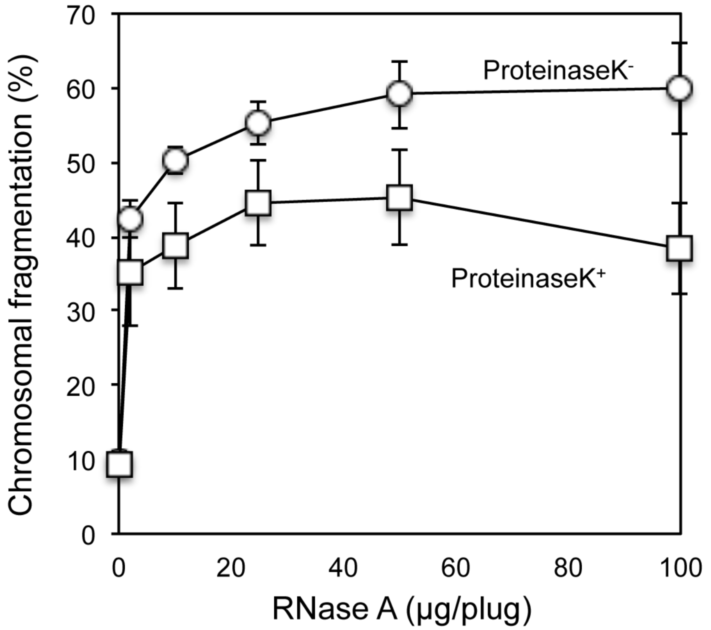

S2 Fig. Effect of proteinase K on RNase dose dependent fragmentation. RiCF in AB1157 when plugs were made with 0, 2, 10, 25, 50 or 100 µg RNase either with or without 25 µg/plug proteinase K. The values presented are means of 3-6 independent assays ± SEM.
