# Supplementary material for: Degradation of RNA during lysis of Escherichia coli cells in agarose plugs breaks the chromosome
Source: PLoS One. 2017 Dec 21;12(12):e0190177. doi: 10.1371/journal.pone.0190177 (PMC5739488; doi:10.1371/journal.pone.0190177)
Supplement: S3 Fig — (PDF) [file pone.0190177.s003.pdf]

S3

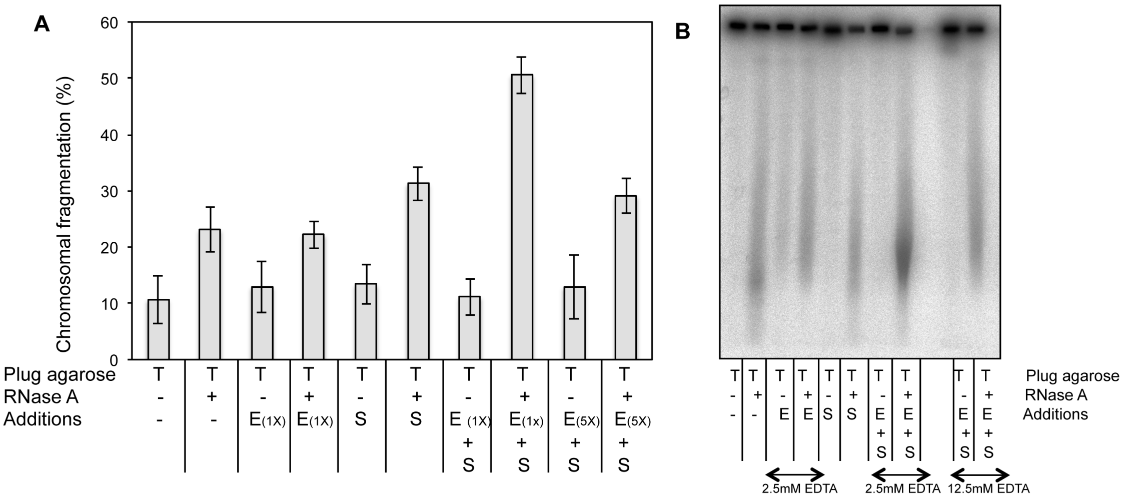

S3 Fig. Effect of sarkosyl and EDTA in plugs on RiCF. Cultures of AB1157 were embedded in presence and absence of RNase in plug agarose containing Tris (50 mM), tris (50 mM) EDTA (5 mM), Tris (50 mM) EDTA (5 mM) sarkosyl (0.2%), or Tris (50 mM) EDTA (25 mM) sarkosyl (0.2%). All plugs were lysed under standard conditions in regular lysis buffer. **(A)** Quantification of fragmentation. The values presented are means of 3-4 independent assays ± SEM. **(B)** A representative radiogram from which data in (A) were derived.
