# Supplementary material for: Degradation of RNA during lysis of Escherichia coli cells in agarose plugs breaks the chromosome
Source: PLoS One. 2017 Dec 21;12(12):e0190177. doi: 10.1371/journal.pone.0190177 (PMC5739488; doi:10.1371/journal.pone.0190177)
Supplement: S4 Fig — (PDF) [file pone.0190177.s004.pdf]

S4

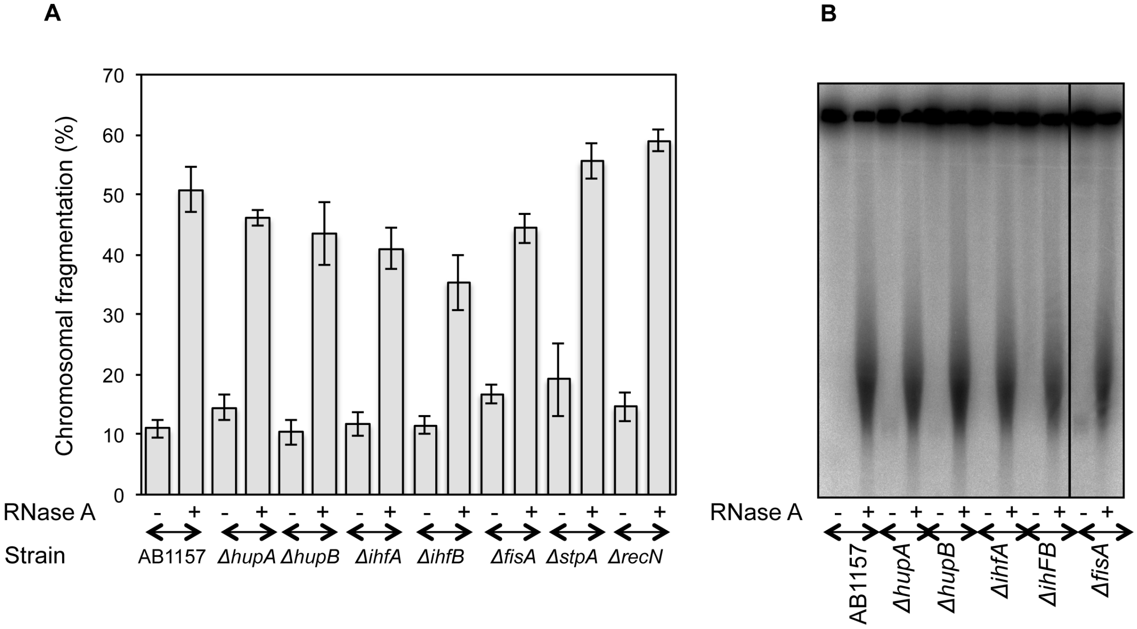

S4 Fig. Effect of NAP mutants on RiCF. (A) Quantification of spontaneous and RNase-induced chromosomal fragmentation in AB1157 and its various NAP mutants after their growth to the same optical density of 0.6. The values presented are means of 3-4 independent assays ± SEM. **(B)** A radiogram showing qualitative fragmentation profiles of some of the mutants quantified in (A).
