# Supplementary material for: Degradation of RNA during lysis of Escherichia coli cells in agarose plugs breaks the chromosome
Source: PLoS One. 2017 Dec 21;12(12):e0190177. doi: 10.1371/journal.pone.0190177 (PMC5739488; doi:10.1371/journal.pone.0190177)
Supplement: S5 Fig — (PDF) [file pone.0190177.s005.pdf]

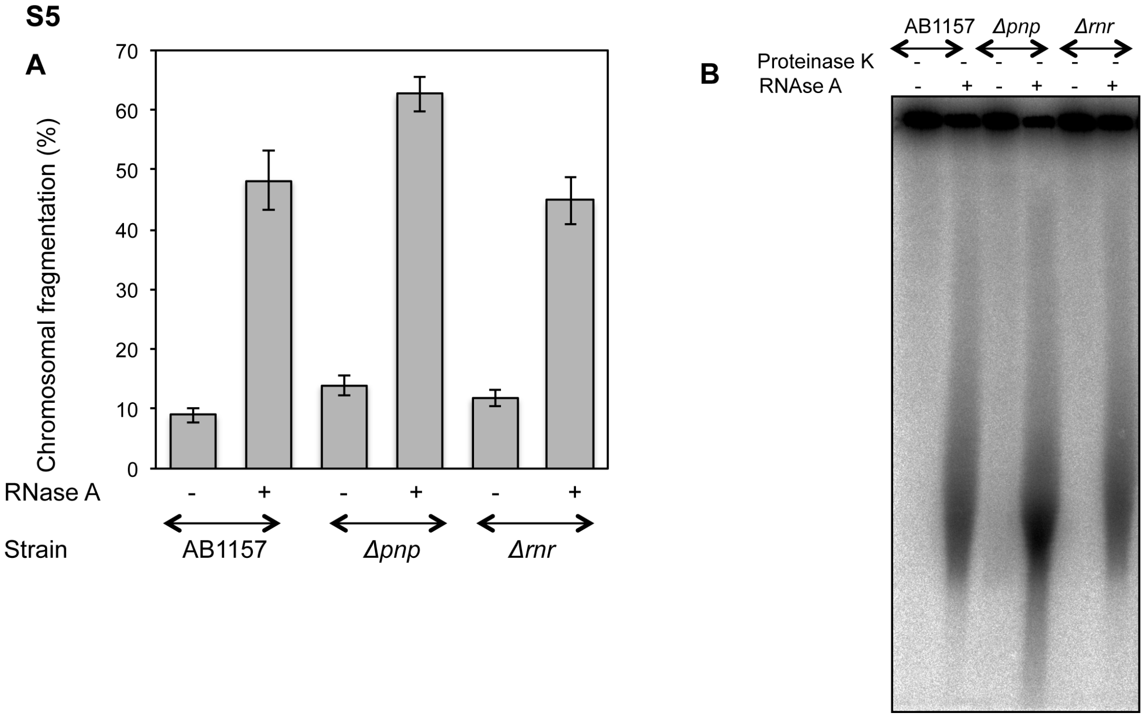

S5 Fig. Effect of deletion of RNA degrading enzymes PNPase and RNase R on RiCF. **(A)** Quantitative comparison of spontaneous and RNase-induced chromosomal fragmentation in AB1157 and its Δ*pnp* and Δ*rnr* derivatives. The values presented are means of 9 independent assays ± SEM. **(B)** A representative radiogram from which data in (A) is derived.
