# Supplementary material for: Degradation of RNA during lysis of Escherichia coli cells in agarose plugs breaks the chromosome
Source: PLoS One. 2017 Dec 21;12(12):e0190177. doi: 10.1371/journal.pone.0190177 (PMC5739488; doi:10.1371/journal.pone.0190177)
Supplement: S6 Fig — (PDF) [file pone.0190177.s006.pdf]

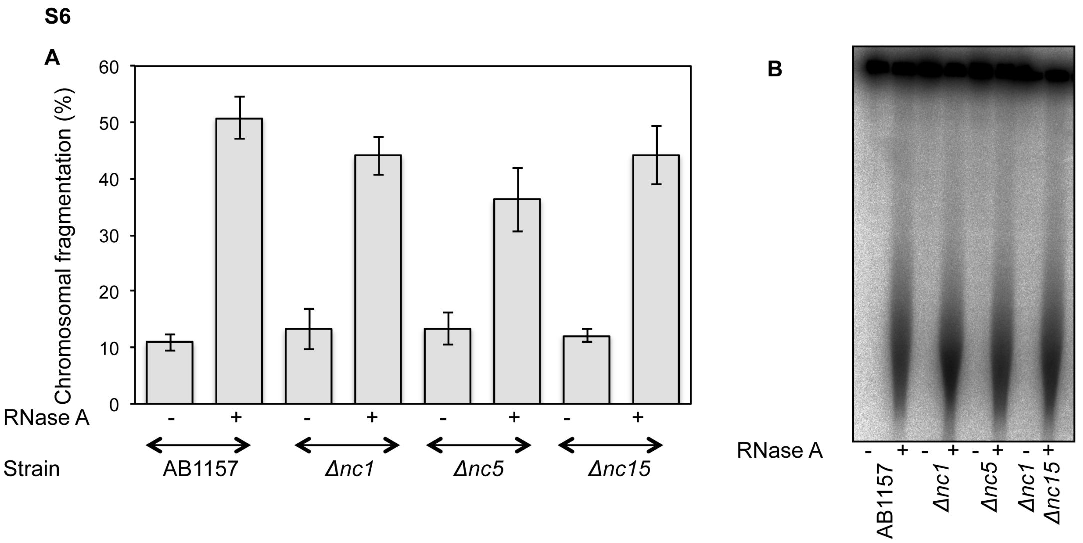

S6 Fig. Effect of deletion of non-coding RNAs *nc1* and *nc5* on RiCF. (A) Quantification of spontaneous and RNase-induced chromosomal fragmentation in AB1157, AB1157 Δ*nc1*, AB1157 Δ*nc5* and AB1157 Δ*nc15*. The values presented are means of 4-8 independent assays ± SEM. **(B)** A representative radiogram from which data in (A) is derived.
