# Supplementary material for: Degradation of RNA during lysis of Escherichia coli cells in agarose plugs breaks the chromosome
Source: PLoS One. 2017 Dec 21;12(12):e0190177. doi: 10.1371/journal.pone.0190177 (PMC5739488; doi:10.1371/journal.pone.0190177)
Supplement: S7 Fig — (PDF) [file pone.0190177.s007.pdf]

S7

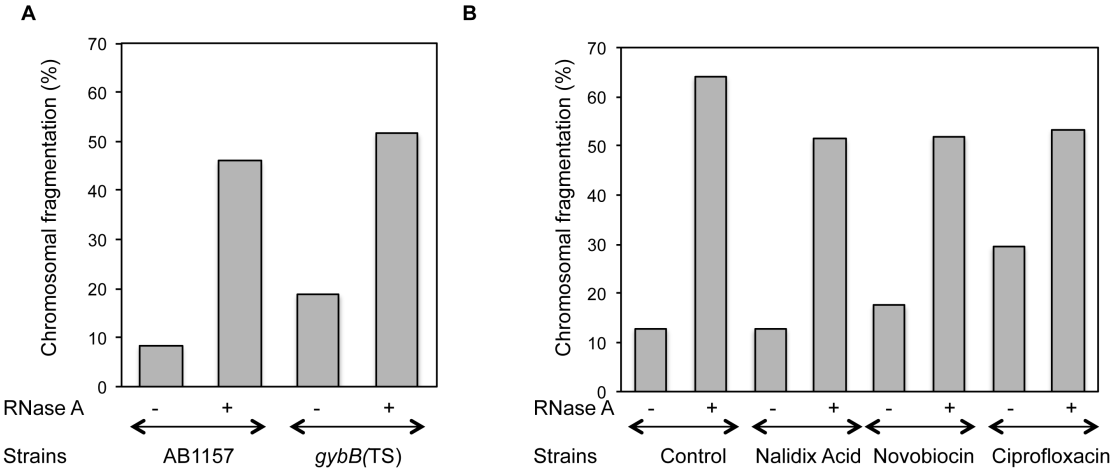

S7 Fig. Effect of inhibition of DNA gyrase on RiCF. (A) AB1157 and AB1157 *gyrB*(TS) were grown at 28°C till OD 0.2 and then transferred to 42°C for 90 minutes before harvesting the cells. Plugs were made in the presence or absence of RNase. **(B)** AB1157 was grown at 37°C until OD 0.6, distributed into four parts and in three parts antibiotics were added to concentrations of: nalidixic acid, 100 µg/ml; novobiocin, 100 µg/ml and ciprofloxacin, 100 ng/ml. All cultures were incubated at 37°C for 10 minutes before making plugs in the presence or absence of RNase. The values presented are means of two assays.
