# Supplementary material for: Degradation of RNA during lysis of Escherichia coli cells in agarose plugs breaks the chromosome
Source: PLoS One. 2017 Dec 21;12(12):e0190177. doi: 10.1371/journal.pone.0190177 (PMC5739488; doi:10.1371/journal.pone.0190177)
Supplement: S8 Fig — (PDF) [file pone.0190177.s008.pdf]

S8

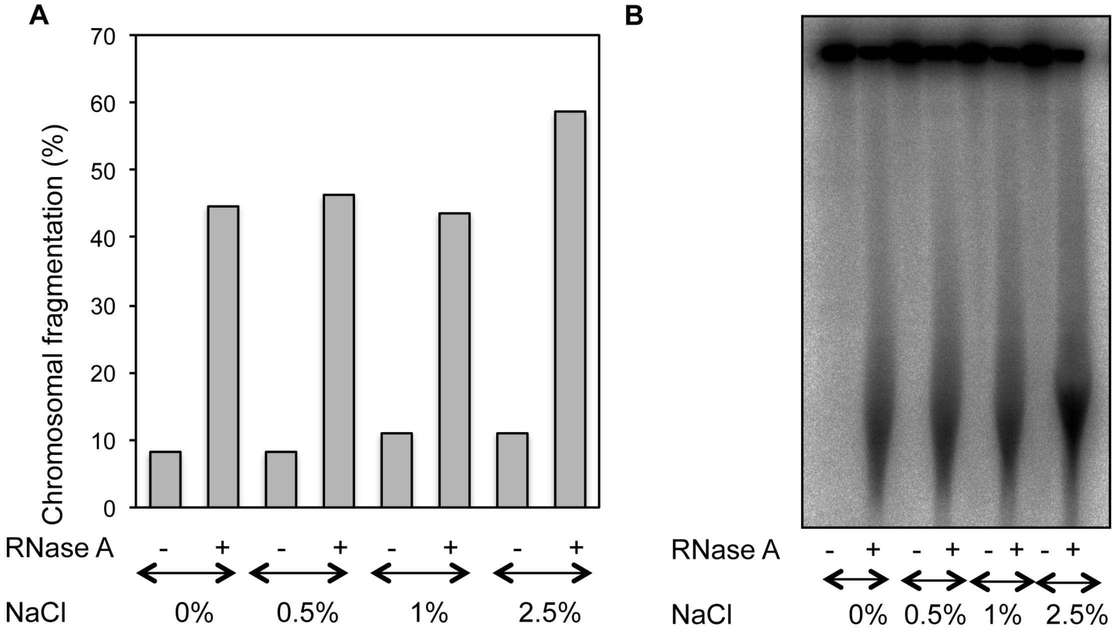

S8 Fig. Effect of osmolarity of growth medium on RiCF. (A) AB1157 was grown at 37°C in LB containing 0, 0.5, 1.0 or 2.5% NaCl. Once the cultures reached the OD 0.6, cells were harvested and made into plugs in the presence or absence of RNase. The values presented are means of two assays. **(B)** A representative radiogram from which data in (A) is derived.
