# Supplementary material for: Degradation of RNA during lysis of Escherichia coli cells in agarose plugs breaks the chromosome
Source: PLoS One. 2017 Dec 21;12(12):e0190177. doi: 10.1371/journal.pone.0190177 (PMC5739488; doi:10.1371/journal.pone.0190177)
Supplement: S9 Fig — (PDF) [file pone.0190177.s009.pdf]

S9

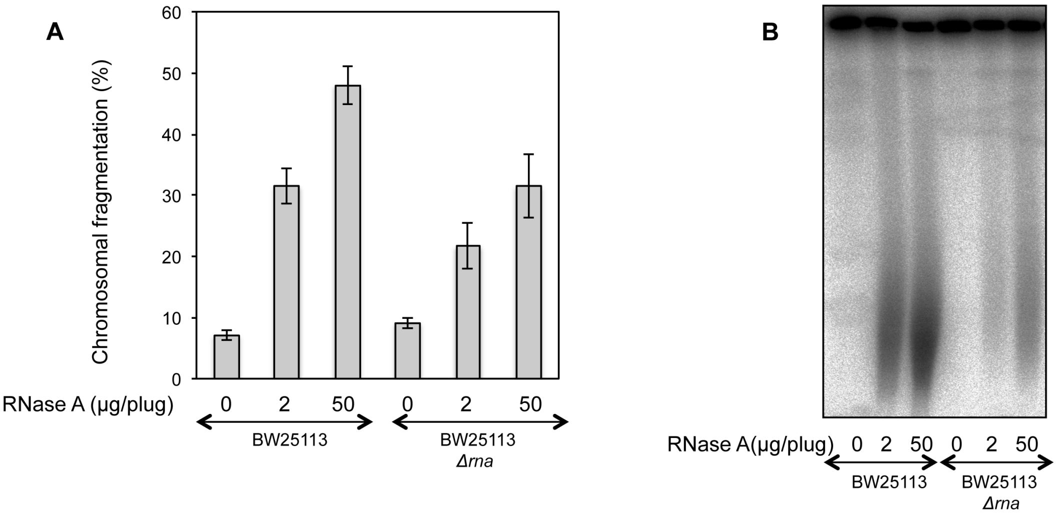

S9 Fig. Effect of deletion of ribonuclease-I on RiCF. (A) Quantitative comparison of spontaneous and RNase-induced chromosomal fragmentation in BW25113 and its Δ*rna* derivative. The plugs were made from 0.6 OD cultures using 0, 2 or 50 µg RNase. The values presented are means of 3 independent assays ± SEM. **(B)** A representative radiogram from which data in (A) is derived.
