# Supplementary material for: Degradation of RNA during lysis of Escherichia coli cells in agarose plugs breaks the chromosome
Source: PLoS One. 2017 Dec 21;12(12):e0190177. doi: 10.1371/journal.pone.0190177 (PMC5739488; doi:10.1371/journal.pone.0190177)
Supplement: S10 Fig — (PDF) [file pone.0190177.s010.pdf]

S10

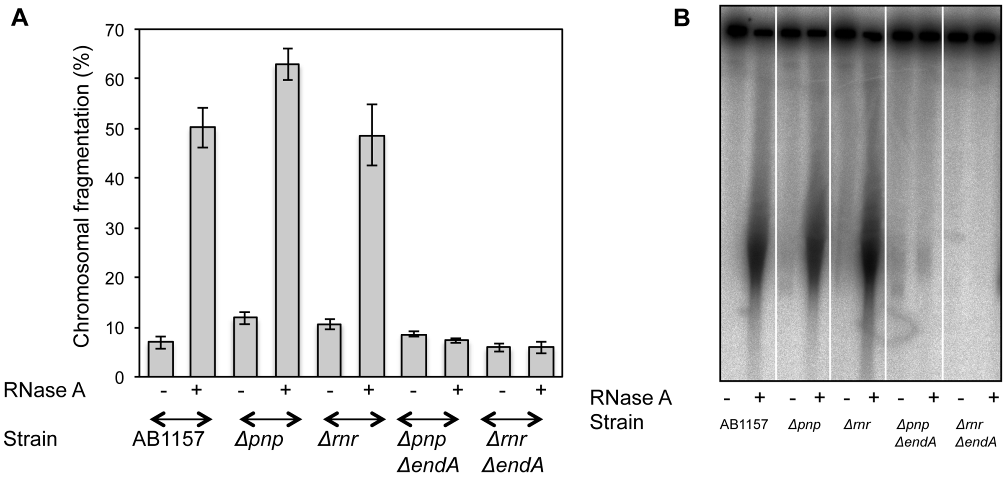

S10 Fig. Effect of endonuclease-I on RiCF of Δ*pnp* and Δ*rnr* mutants. (A) Quantitative comparison of spontaneous and RNase-induced chromosomal fragmentation. All strains were grown to the same final OD of ca. 0.6 and made into plugs in presence or absence of RNase. Data points are means of 3-4 independent assays ± SEM. **(B)** A representative radiogram from which data in (A) is derived.
