# Supplementary material for: Degradation of RNA during lysis of Escherichia coli cells in agarose plugs breaks the chromosome
Source: PLoS One. 2017 Dec 21;12(12):e0190177. doi: 10.1371/journal.pone.0190177 (PMC5739488; doi:10.1371/journal.pone.0190177)
Supplement: S11 Fig — (PDF) [file pone.0190177.s011.pdf]

S11

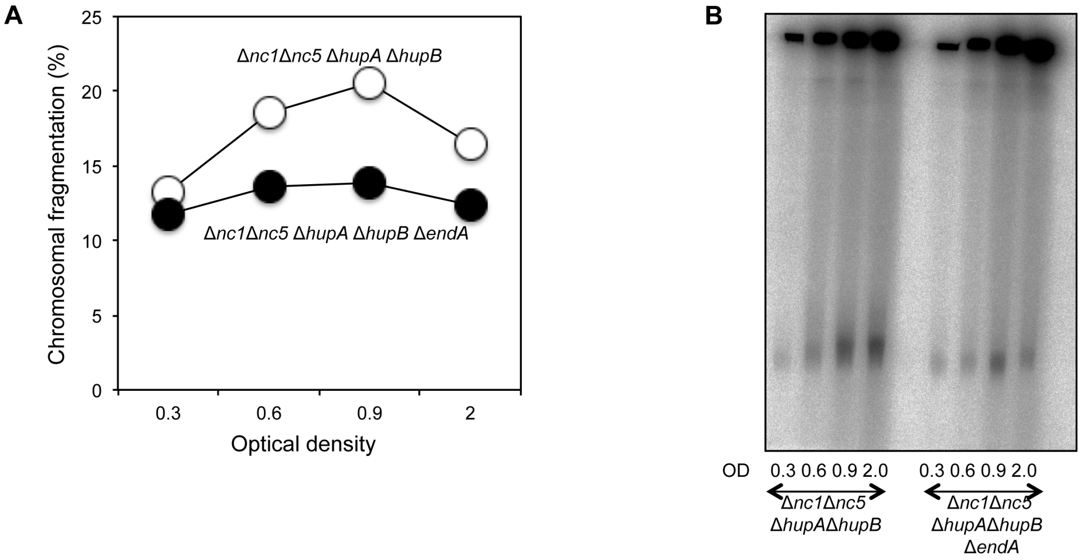

S11 Fig. Kinetics of spontaneous fragmentation. (A) Comparison of spontaneous fragmentation between AB1157 Δ*nc1* Δ*nc5* Δ*hupA* Δ*hupB* and AB1157 Δ*nc1* Δ*nc5* Δ*hupA* Δ*hupB* Δ*endA* mutants. Both strains were grown to various ODs and plugs were made in the absence of RNase. **(B)** The radiogram from which data in (A) is derived.
