# Supplementary material for: Degradation of RNA during lysis of Escherichia coli cells in agarose plugs breaks the chromosome
Source: PLoS One. 2017 Dec 21;12(12):e0190177. doi: 10.1371/journal.pone.0190177 (PMC5739488; doi:10.1371/journal.pone.0190177)
Supplement: S12 Fig — (PDF) [file pone.0190177.s012.pdf]

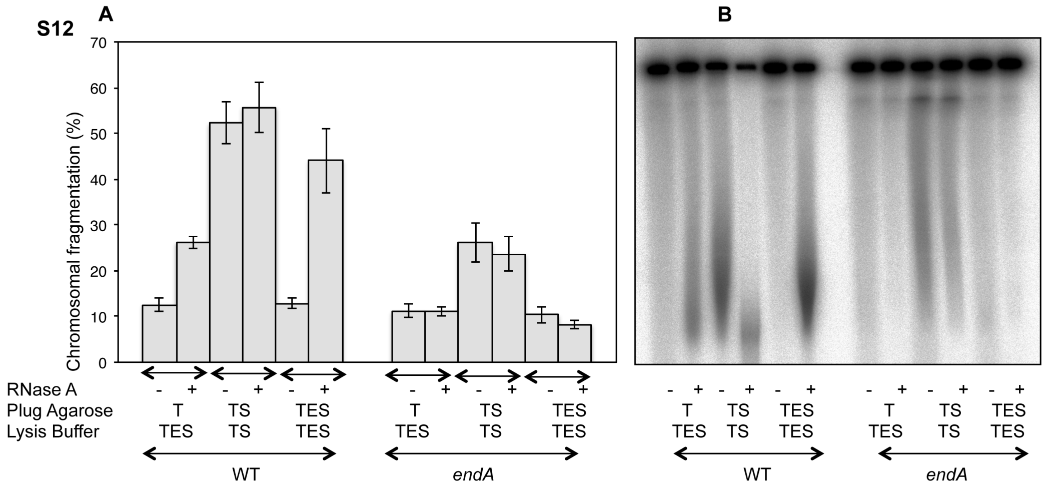

S12 Fig. Plug lysis in EDTA-less lysis buffer causes spontaneous fragmentation. (A) Comparison of spontaneous fragmentation and RiCF when AB1157 cells were suspended in TE and made into plugs in lysis agarose either with or without EDTA. Plugs were then incubated in lysis buffer containing no or 25mM EDTA and electrophoresed for fragmentation analysis. The data points are means of 3-5 independent experiments ± SEM. **(B)** Radiogram from one of the experiment from which data in (A) is derived. T, Tris; TS, Tris Sarkosyl; TES, Tris EDTA Sarkosyl.
