# Supplementary material for: Degradation of RNA during lysis of Escherichia coli cells in agarose plugs breaks the chromosome
Source: PLoS One. 2017 Dec 21;12(12):e0190177. doi: 10.1371/journal.pone.0190177 (PMC5739488; doi:10.1371/journal.pone.0190177)
Supplement: S13 Fig — (PDF) [file pone.0190177.s013.pdf]

S13

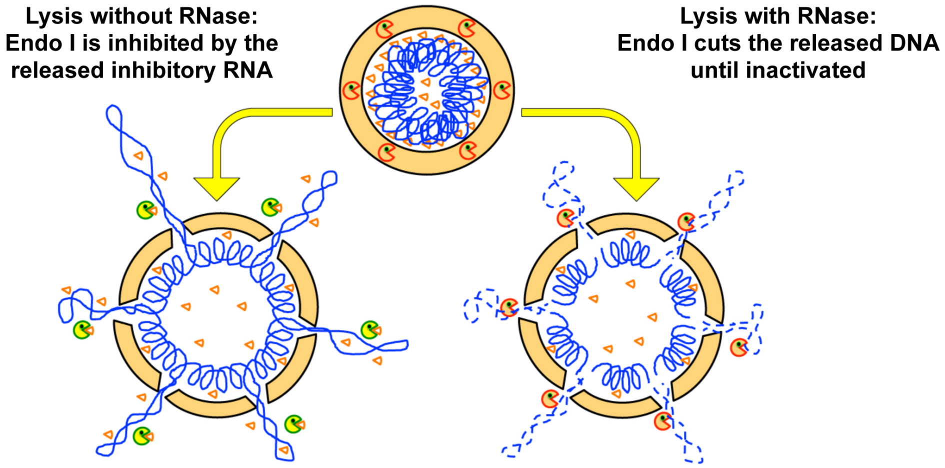

S13 Fig. Incomplete degradation of chromosomes during RiCF could be due to contact limitation. During cell lysis, parts of the chromosomes are pushed through the disrupted cell membrane. Since the physical movement of macromolecules is not free in agarose plugs, only those parts of the chromosomes which come in contact with the activated endonuclease I are degraded before endonuclease I becomes inactivated. This leads to the generation of chromosomal fragments (short spirals within the lysing cell in the right) detected in PFGE.
